# Tools and approaches for mapping Marine Animal Forests: A practical overview for researchers and conservationists

**DOI:** 10.12688/openreseurope.20823.2

**Published:** 2026-02-14

**Authors:** Laurence H. De Clippele, Ricardo Aguilar, Miquel Canals, Giovanni Chimienti, Laura Martín-García, Iliyan Kotsev, Bogdan Prodanov, Dimitris Poursanidis, Beatriz Vinha

**Affiliations:** 1 School of Biodiversity One Health and Veterinary Medicine, University of Glasgow College of Medical Veterinary and Life Sciences, Glasgow, Scotland, G12 8QQ, UK; 2 Oceana, Madrid, Spain; 3 Sustainable Blue Economy Chair and CRG Marine Geosciences, Department of Earth and Ocean Dynamics, University of Barcelona, Barcelona, 08028, Spain; 4 Reial Acadèmia de Ciències i Arts de Barcelona (RACAB), Barcelona, 08002, Spain; 5 Institut d’Estudis Catalans (IEC), Barcelona, 08001, Spain; 6 Department of Biosciences, University of Bari Aldo Moro, Bari, Italy; 7 CoNISMa, Rome, Italy; 8 Centro Oceanográfico de Canarias (COC-IEO), CSIC, Santa Cruz de Tenerife, Canary Islands, 38180, Spain; 9 Coastal Zone Dynamics Department, Institute of Oceanology, Bulgarian Academy of Sciences, Varna, Bulgaria; 10 Geotectonics and Regional Geology Department, Geological Institute, Bulgarian Academy of Sciences, Sofia, Bulgaria; 11 Foundation for Research and Technology—Hellas (FORTH), Institute of Applied and Computational Mathematics, N. Plastira 100, Vassilika Vouton, Heraklion, 70013, Greece; 12 Departament de Biologia Evolutiva, Universitat de Barcelona, Barcelona, Spain; 13 Institut de Recerca de La Biodiversitat (IRBio), Universitat de Barcelona, Barcelona, Spain

**Keywords:** Marine Animal Forests, habitat mapping, modelling, multibeam, side scan sonar, satellites, drones

## Abstract

Mapping marine animal forests (MAFs) is essential for understanding complex benthic ecosystems and supporting their conservation and management. This review provides a comprehensive overview of the key aspects of MAFs that can be mapped, focusing on both biological and substrate data. We summarise the diverse platforms and technologies used to collect relevant data, including space, air, and sea-based mapping tools. In addition, we highlight the software tools, open-source databases, and modelling approaches that enable researchers to analyse and map MAFs effectively. The modelling approaches include unsupervised mapping techniques, geomorphological classification, species distribution modelling, biomass distribution modelling, and community distribution modelling. Given the variability in habitat types, depths, and spatial scales, we discuss how geophysical data often serve as proxies for environmental conditions that influence the distribution of species and substrates. The increasing use of machine learning and advanced modelling techniques is also addressed as a means to overcome gaps in biological and substrate data and achieve comprehensive spatial predictions. Finally, we present two practical decision-support flow charts to help guide researchers and practitioners in selecting appropriate mapping tools and modelling approaches based on specific project objectives, environmental settings, and data availability. This review offers a practical toolbox for marine scientists, conservationists, and managers aiming to map and understand the structure and distribution of MAFs more effectively.

## Introduction

### The importance of mapping marine animal forests

Marine animal forests (MAFs) are marine benthic habitats dominated by a three-dimensional structure formed by megabenthic invertebrates (> 1cm) such as anthozoans, sponges, sea pens, ascidians, tubeworms, bryozoans or hydrozoans ( Rossi, 2013; Rossi *et al.*, 2017). MAFs are key ecosystems that, through their structural complexity, are used by associated organisms as a habitat, refuge, nursery and for food ( Cau *et al.*, 2021; De Clippele *et al.*, 2025). According to the EU Habitat Directive, the term habitat refers to an environment defined by specific abiotic and biotic factors, in which a species lives at any stage of its biological cycle (Council Directive 92/43/EEC). From a biological perspective, benthic habitat might more simply be defined as the place where an organism lives with associated physical descriptors of that place, such as seafloor morphology, geological substrate or oceanography ( Lamarche *et al.*, 2016).

In the field of marine ecology and biogeography, mapping MAFs is important to help improve the knowledge of species and habitat distributions, habitat complexity, ecological processes, and the structure and dynamics of marine ecosystems ( Brown *et al.*, 2011a). This information is valuable for identifying critical habitats, assessing human impacts on marine ecosystems, and supporting spatial planning and management for the conservation and protection of marine resources ( Harris & Baker, 2020; Lamarche *et al.*, 2016). There are numerous examples of the importance of mapping, from the discovery of topographic structures of critical ecosystems (e.g. Diesing & Thorsnes, 2018; Gafeira *et al.*, 2018), to understanding patterns in biodiversity and species distribution (e.g. Vinha *et al.*, 2024), from ecosystem functioning ( De Clippele *et al.*, 2021a; De Clippele *et al.*, 2021b; Greiffenhagen *et al.*, 2024) and to provide guidance for management, conservation plans, and policy decisions ( Davies *et al.*, 2015; Mumby & Harborne; Ramiro-Sánchez *et al.*, 2019). While underwater mapping is a crucial field with wide-ranging applications and significant importance across various domains - from exploration, scientific research, and marine management to maritime navigation or infrastructure development – currently (July 2025), 27.3% of the ocean has been explored while the majority (i.e. 72.7%) remains unmapped ( Mayer *et al.*, 2018; Seabed 2030.org), with less than 5% being mapped to a resolution similar to land-based maps ( Lim *et al.*, 2021). This leaves our understanding of what lies beneath the surface incomplete and fragmented.

**Figure 1.**
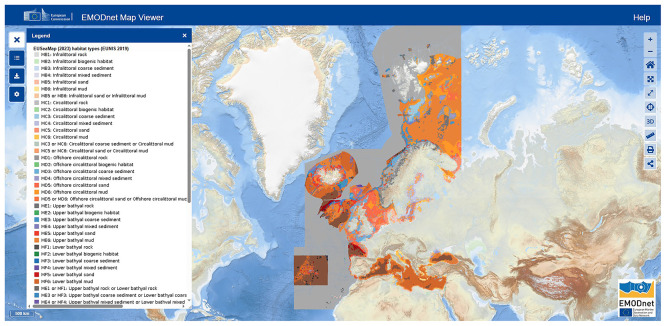
Map of predicted seabed habitat types (EUNIS 2019 classification) across European waters as visualized in the EMODnet Map Viewer ( Vasquez *et al.*, 2021). Color-coded categories correspond to habitat types listed in the legend. Source: European Marine Observation and Data Network (EMODnet).

Currently, the EUSeaMap ( Figure 1) is the only pan-European cartographic product that provides a standardised transboundary overview of the spatial distribution of seabed habitats across Europe ( Vasquez *et al.*, 2021). The European Nature Information System (EUNIS) categories have been utilised by the European Commission's marine data service, the European Marine Observation and Data Network (EMODNET), to develop this transnational, broad-scale seabed habitat map (i.e., EUSeaMap). EUNIS habitat classification encompasses all habitats, ranging from natural to artificial, including terrestrial, freshwater, and marine environments. The latest EUNIS version describes more than 1,600 marine benthic habitats, organised into seven main biological zones: littoral, infralittoral, shallow circalittoral, deep circalittoral, upper bathyal, lower bathyal, and abyssal. The EUNIS habitat type A5.6, the sublittoral biogenic reefs include several MAFs such as polychaeta reefs, bivalve reefs and cold-water coral reefs, while type A6.62 is a deep-sea sponge aggregation. The continuous advancement of technology and knowledge of the seabed worldwide results in constant updates to the EUNIS classification and the EMODnet cartographic products.

Here, we provide an overview of the aspects (i.e., species or substrate data) of a MAF that can be mapped, along with the platforms and modelling tools available to do so. Over the last few decades, advanced underwater mapping technology has significantly enhanced our understanding of the seafloor environment and the biodiversity associated with MAFs. To date, most underwater maps display information on the local geomorphology, environmental conditions or biological data ( Kavanaugh *et al.*, 2016). Depending on the depth, area, time period, and the resolution at which a MAF is to be mapped, different mapping platforms and tools can be used. To map biological data, geophysical data (e.g. depth, rugosity, slope, eastness, northness, bathymetric positioning index, backscatter) are often used, as proxies for oceanographic conditions (e.g. food supply, temperature, current speed), and are integrated with biological data (e.g. species presence) extracted from videos, images, or physical samples. As biological data are often limited, species and habitat distribution modelling approaches are increasingly being used to develop full-coverage MAF maps, i.e. without spatial data gaps.

### Types of data: biological and substrate

To confirm the presence of and comprehensively understand patterns in the spatial distribution of a MAF habitat, it is essential to gather data on the presence of a MAF habitat-forming species and the associated substrate. This data is then often analysed alongside environmental data (e.g., terrain variables, temperature, salinity, current speed and orientation) to understand what drives spatial patterns.

***Species.*** The presence of a MAF habitat-forming species can be established using imaging tools (photography/videography), active acoustic (i.e. sonar) or physical samples (e.g. De Clippele *et al.*, 2017; Huvenne *et al.*, 2003; Piechaud & Howell, 2022). Thanks to the development of remotely operated vehicles (ROVs), autonomous underwater vehicles (AUVs), submersibles, and drones, the spatial coverage and amount of image data of MAFs have drastically increased ( Clark *et al*., 2024; Petillot *et al*., 2019). To increase the accuracy, efficiency and transparency of image and video annotations, several open-access platforms have been developed. Examples of open-source platforms for annotating video and images include BIIGLE ( Langenkämper *et al.*, 2017), Video Annotation and Reference System (VARS) ( Schlining & Stout, 2006), Squidle+; ( https://squidle.org) and PAPARA(ZZ)I ( Marcon & Purser, 2017).

One of the challenges of using approaches based on video and image analysis is that high-resolution taxonomic identification is not always possible. However, there are different standardised annotation protocols for image-based identifications that can be followed. Some of these protocols include the CATAMI classification scheme ( Althaus *et al.*, 2015), SmarTaR-ID ( Howell *et al.*, 2019) or the use of Open nomenclature (ON) signs ( Horton *et al.*, 2021). Instead of collecting one’s own data, it is possible to source pre-collected data from databases such as: Ocean Biogeographic Information System (OBIS) ( https://obis.org), the Global Biodiversity Information Facility (GBIF) ( https://www.gbif.org), NOAA’s Deep-sea Coral Portal ( https://deepseacoraldata.noaa.gov), UNEP Global Distribution of Cold-water corals ( Freiwald *et al.*, 2021; https://resources.unep-wcmc.org/products/fb9b160602e84a139ffc4fc16cf74bfc) and Online data repositories such as PANGAEA ( https://pangaea.de), Dryad ( https://datadryad.org), Zenodo ( https://zenodo.org/) and Figshare ( https://figshare.com). Note that while some databases included raw data, some may contain modelled or inferred data and would therefore need to be interpreted with care. When searching for relevant data, researchers should review both the repository entries and the corresponding publications, as data are frequently linked or described within related papers.

Another challenge is related to the analysis of large image datasets. Deep learning approaches offers a solution, such as the use of convolutional neural networks, are becoming increasingly accessible for benthic ecologists. Examples of user-friendly tools are YOLO ( Piechaud & Howell, 2022), RootPainter ( Clark *et al.*, 2024), and MAIA in BIIGLE ( Cuvelier *et al.*, 2024).

While imagery collected by ROVs, AUVs and drones provides valuable non-invasive insights into marine habitats and species distributions, physical biological sampling remains essential for accurate species identification. Many marine organisms, particularly invertebrates, exhibit morphological similarities that are difficult to distinguish solely through imagery. Physical samples can e.g. be collected with ROVs, trawls, grab-and boxcore samplers, and enable detailed taxonomic analysis, including microscopic and genetic examination, which is crucial for confirming species identity, identifying cryptic species, and refining image-based identification protocols. Integrating physical sampling with visual data enhances the reliability and ecological relevance of biodiversity assessments, especially in deep-sea and poorly studied marine environments

***Substrate.*** The seabed is covered by a diverse range of substrates that play a crucial role in marine ecosystems, sediment transport, and seabed stability. These substrates influence benthic habitats ( Romoth *et al.*, 2023), biogeochemical cycles ( Herbert, 1999; Microbial Ecology *et al.*) and human activities such as fisheries ( Langton & Auster, 1999), offshore construction ( Gerwik Jr, 2007), and environmental monitoring ( Rice *et al.*, 2012). Mapping and classifying seabed substrates are fundamental for marine spatial planning and conservation efforts ( Boero *et al.*, 2016; Papenmeier *et al.*, 2020). Seabed substrates can be classified based on their composition and formation processes. The primary categories include sediment-based classification, grain size and texture and sediment composition.

**Figure 2.**
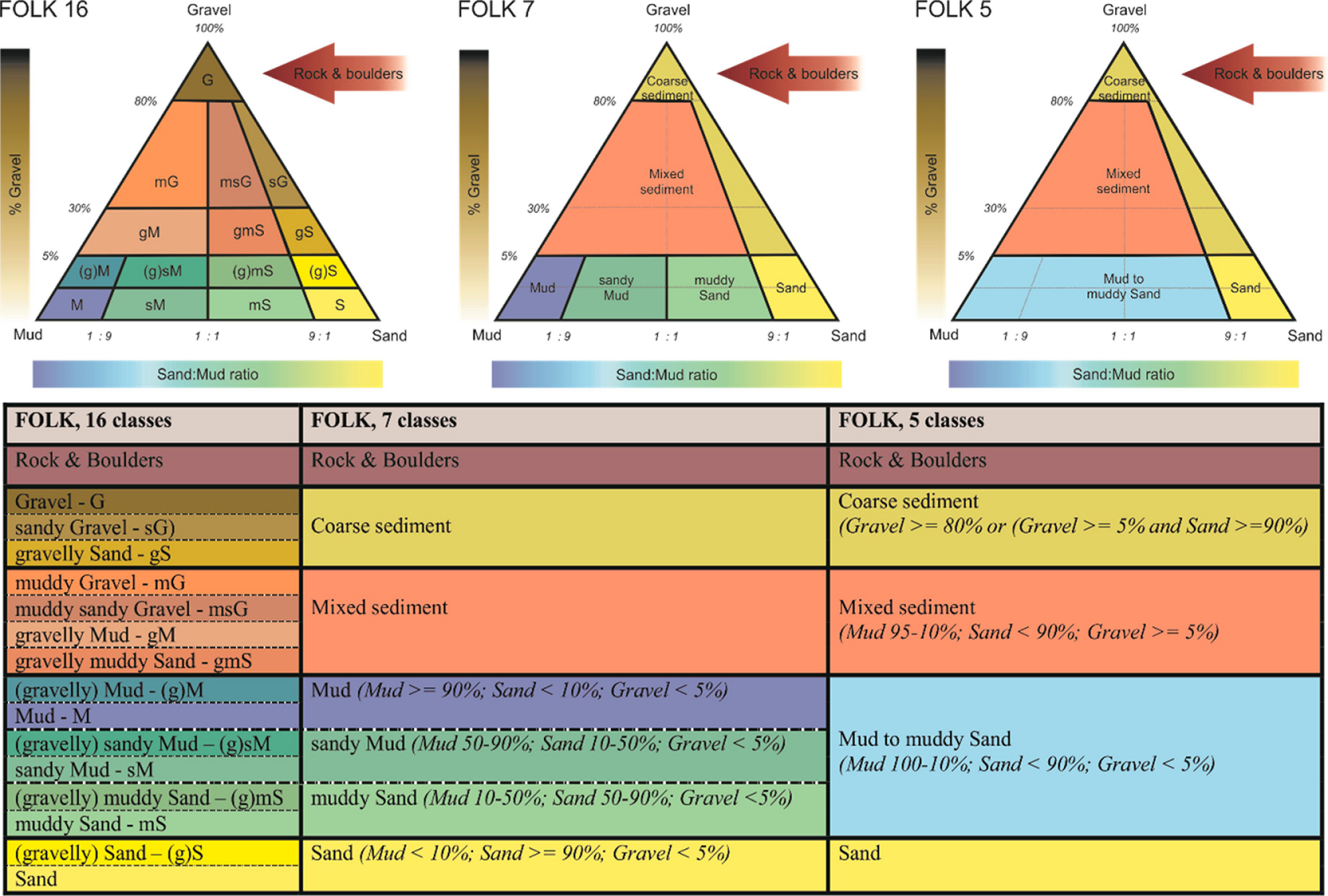
The Folk sediment triangle and the hierarchy of Folk classification (16, 7 and 5classes, plus an additional class “Rock and boulders,” indicated by the arrow) used in the EMODnet Geology project ( Kaskela *et al.*, 2019).

A widely used classification system is based on sediment grain size, following the Folk (1954) ( Figure 2) and Wentworth (1922), and Udden (1914) scales. The primary categories include mud (<63 µm) (i.e. composed of clay and silt fractions), sand (63 µm - 2 mm) (i.e. further divided into fine, medium, and coarse sand) and gravel (>2 mm) (i.e. includes granules, pebbles, cobbles, and boulders (> 64 mm). Within the Folk classification system, there are different systems (e.g. folk 16-class system, folk-7 class system) with the main difference between these classifications lying in the proportion of sediment fractions ( Figure 2). For example, sediments with more than 90% sand are classified as sand, whereas those with 50–90% sand mixed with finer particles fall into the muddy sand category. Similarly, substrates with at least 30% gravel are categorised as gravelly sediments, while those with less gravel but more sand and mud are classified as mixed sediments. These classifications support habitat classification frameworks such as the EUSeaMap, 2023 Broad-Scale Predictive Habitat Map for Europe ( Vasquez *et al.*, 2021) and the EMODnet Geology Seabed Substrate classification ( https://emodnet.ec.europa.eu/en/geology).

Grain size is a key determinant of sediment transport, seabed stability, and habitat suitability. The Wentworth (1922) classifies sediments based on particle size, influencing sediment permeability and organic matter retention: clay (<0.0039 mm), silt (0.0039 mm - 0.0625 mm), sand (0.0625 mm - 2 mm), granule (2 mm - 4 mm), pebble (4 mm - 64 mm), cobble (64 mm - 256 mm) and boulder (>256 mm). Larger grains such as gravel and boulders are typically found in high-energy environments where strong currents or waves transport heavier materials. Conversely, finer grains, such as silt and clay, settle in low-energy environments, where calm conditions allow very small particles to accumulate.

The relationship between grain size and sedimentary processes also extends beyond physical classification, linking substrate characteristics to broader ecological and biogeochemical functions of the seabed. Beyond its geomorphological relevance, grain size plays a key role in carbon tracking and climate-smart spatial planning. Fine-grained sediments, such as silt and clay, are recognised as effective sinks for organic carbon due to their high surface area, low permeability and capacity to protect organic matter from remineralisation ( Bao *et al.*, 2019; Guo *et al.*, 2021; Müller *et al.*, 2025). In contrast, coarse sediments are typically associated with high hydrodynamic energy and limited carbon retention capacity ( Dahl *et al.*, 2016). Mapping depositional environments dominated by fine-grained sediments is therefore essential for identifying natural carbon sinks and understanding the spatial variability of organic carbon burial ( Legge *et al.*, 2020; Wang *et al.*, 2024). Integrating grain-size and carbon-content data within marine habitat mapping frameworks enables a more comprehensive assessment of seabed functions, supporting climate-smart spatial planning and the prioritisation of areas contributing to long-term carbon sequestration and ecosystem resilience ( Müller *et al.*, 2025; Zhang *et al.*, 2024).

Grain-size analysis represents a fundamental step in characterising marine substrates, as engineering projects and habitat-mapping tasks require high analytical precision and reliability in determining sediment composition and texture. Such analyses are conducted in specialised sediment laboratories, where standardised methodologies and controlled conditions ensure consistency of results. Laboratory techniques commonly include wet and dry sieving for coarser fractions, hydrometer and pipette analyses for fine-grained particles, and laser granulometry for detailed particle-size spectra. The processing, statistical treatment, and graphical visualisation of these datasets are frequently performed using the Excel-based software GRADISTAT, developed by Blott and Pye (2001), which enables standardised and reproducible sediment classification across marine and terrestrial environments. These laboratory-based approaches provide the quantitative foundation for accurate interpretation of depositional dynamics, substrate heterogeneity, and sediment-associated biogeochemical processes.

Beyond grain size, seabed sediments are also classified based on their origin and composition ( Chester & Jickells, 2012; Knauss & Garfield, 2016; Seibold & Wolfgang, 2017, etc.). Terrigenous sediments are derived from land-based sources, transported to the ocean by rivers, wind, or glaciers. They are the most abundant and include sand, silt, and clay materials. Biogenous sediments are composed of the hard parts of marine organisms, such as shells and skeletons. When these materials constitute at least 30% of the sediment, they are referred to as "oozes". Hydrogenous sediments are formed from the precipitation of dissolved minerals directly from seawater, often found near hydrothermal vents.

Seabed substrate classification plays a significant role in various marine applications. In marine spatial planning it can provide essential data for marine resource management and offshore development projects ( Propp *et al.*, 2016). In MAF mapping, it supports ecosystem conservation efforts and biodiversity assessments ( Boero *et al.*, 2016). For sediment transport studies, it helps predict seabed stability and erosion patterns ( Davies & Thorne, 2008). Fisheries and offshore construction can use this information to decide on how their infrastructure may impact the presence of existing or the formation of new habitats ( Boero *et al.*, 2016). Knowledge of the substrate is also essential to understand how the substrate composition and e.g. coastal flood risk, may change as a consequence of climate change and human activities ( Trifonova *et al.*, 2015).

International and national databases compile seabed sediment data to provide standardised information. A key example is the EMODnet Geology Seabed Substrate Database ( https://emodnet.ec.europa.eu/en/geology), which provides seabed substrate data for European seas based on modelled data.

## Tools and technologies

**Figure 3.**
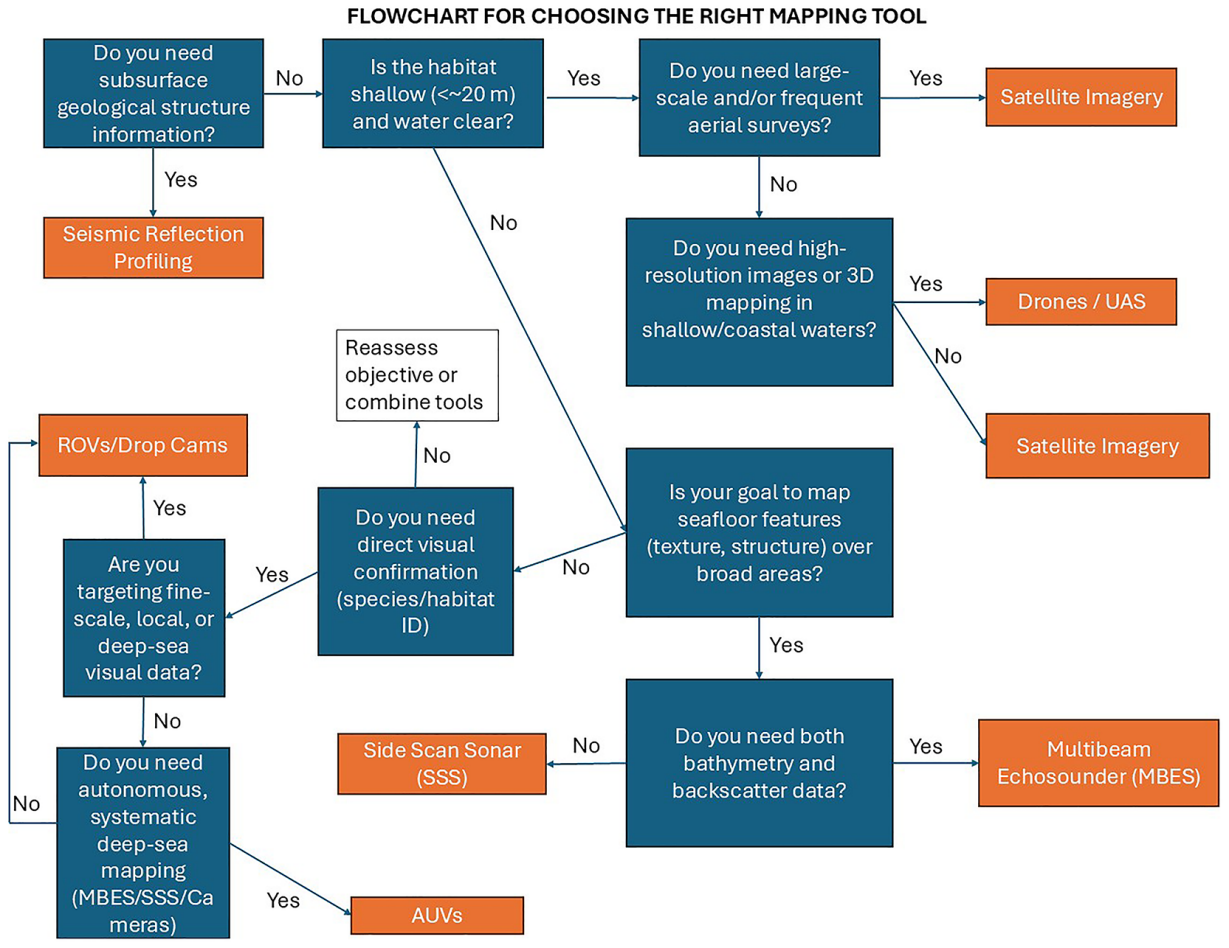
Flowchart to help a user decide which tool to use.

To map MAFs a variety of mapping platforms can be used, operating from space, the air, or the sea ( Kearns & Breman, 2010; Li *et al.*, 2023, and references therein) ( Figure 3). The choice of platform will depend on the habitat’s depth (< or > 20 m), the size of the area that is to be mapped (m ^2^, 10s or 100s km ^2^), and the resolution needed (centimetre to kilometre scale) ( Table 1). A flowchart is provided in Figure 3 to help a user decide on what tool to use ( Figure 3).

**Table 1.** Overview of the pro’s and con’s associated with using each of the tools. Along with examples of publications that used each of the tools.

Tool	Pros	Cons	Examples
Space-based instruments (satellite)	- Suitable for shallow habitats - Large-scale coverage - Useful for shallow, clear waters - Cost-effective for broad surveys - Suitable for long-term monitoring	- Limited to shallow depths (up to ~20 m) as image quality impacted by surface reflections, waves, lighting and turbidity - Poor resolution for fine-scale habitat features - Weather and water clarity dependent - When high resolution data are required, only commercial data is available	Hedley *et al.*, 2018; Lyons *et al.*, 2024
Air-based instruments and unmanned aerial systems (UAS) (drones)	- Suitable for shallow habitats - less affected by clouds vs satellites - High-resolution in shallow/coastal waters in comparison to satellite/airborne methods (up to mm and cm scale) - Enables high resolution photogrammetry, thermal/IR imaging and LiDARgrammetry - Flexible deployment in relation to lighting/weather - Real-time data acquisition - Rapid coverage of large areas - Enhanced accuracy via GCPs and georeferencing - Supports habitat classification with machine learning/AI models	- Limited to shallow depths (up to ~20 m) as image quality impacted by surface reflections, waves, lighting and turbidity - Requires advanced sensors and optimised flight planning - Short data collection timeframe (<10 years), lack of historical/baseline data pre-2000s - Often descriptive rather than comparative analyses - Unusable in air navigation restricted areas.	Casella *et al.*, 2017; Chirayath & Earle, 2016; Collin *et al.*, 2008; Monteiro *et al.*, 2021; Nababan *et al.*, 2021; Ventura *et al.*, 2018; Ventura *et al.*, 2023
Side scan sonar (SSS)	- Suitable for shallow and deep-sea habitats - Effective for detecting habitat structure and texture over wide areas - High spatial resolution for surface features (cm–dm scale, depending on frequency and towfish altitude) - Excellent for mapping large areas - Backscatter SSS data useful for seafloor sediment classification and detecting roughness changes linked to epi- /infaunal assemblages - Dual frequencies could be used simultaneously (vs. resolution). - Allows for mosaicking of individual sonographs	- Can be difficult to interpret and requires expertise - Limited penetration depth - Often needs to be combined with other methods, e.g. for subsurface investigations - Highly dependent on sea state. data acquisition may be affected by environmental factors (e.g., currents, waves, bubbles in the water column, etc.) - Doesn’t provide direct bathymetry information.	Blondel, 2009; Brown *et al.*, 2011a; Jordan *et al.*, 2010; Propp *et al.*, 2016; Rusmadi & Hasan, 2020; Schimel *et al.*, 2010; Switzer *et al.*, 2020; Waddington & Hart, 2003
Seismic reflection profiling	- Suitable for shallow and deep-sea habitats - Subsurface profile, penetrates and images soft substrata - Sediment stratigraphy - Hull-mounted options usable in parallel with multibeam	- Disturbing for fauna - Hard and gassy substrates cannot be penetrated nor imaged - Could result in interferences with other acoustic instruments if operated simultaneously	Angeletti *et al.*, 2019; Fakiris *et al.*, 2018; Wu *et al.*, 2020
Multibeam bathymetry echosounder (MBES)	- Suitable for shallow and deep-sea habitats - Can produce both high-resolution bathymetry and backscatter - Can be used for terrain deriviates - Effective for mapping topographic complexity - Full spatial coverage - Yields large sets of useful derivative maps and other products - Useful for detecting and characterising large morphological features in deep-sea areas.	- Expensive equipment and operation - Can only be used for indirect biological interpretation - Data quality depending on sea state - Requires silent platforms for optimal data quality - When distance between MBES and seafloor is large, the resolution is usually lower compared to when the distance is smaller (e.g. in shallow habitats or when MBES is attached to ROV/AUV (see below)	Brown *et al.*, 2011a; Cui *et al.*, 2021; Micallef *et al.*, 2012; Schimel *et al.*, 2010
Remotely operated vehicles (ROVs) and drop cams	- Suitable for remote and deep-sea habitats - Direct visual confirmation of species and habitat - High-resolution imagery and video - Facilitates fine-scale mapping, allowing the detailed investigation of habitat heterogeneity at local scales. - Can carry MBES, side scan sonar	- Limited spatial coverage - Time-consuming and expensive - Requires expert species knowledge but often results in low resolution of taxonomic identifications. - High annotation effort to process video and images. AI tools are available, but they are still being improved.	( De Clippele *et al.*, 2017; Martín-García *et al.*, 2022; Price *et al.*, 2019; Robert *et al.*, 2016)
Autonomous Underwater Vehicles (AUVs)	- Suitable for remote and deep-sea habitats - Can carry MBES, side scan sonar, or cameras; ideal for systematic surveys of MAFs.	- Autonomous operation reduces ship time but requires complex planning and data handling. - Can be more challenging to use in more complex habitats due to risk of collisions	( Bodenmann *et al.*, 2023; Williams *et al.*, 2010; Zhang *et al.*, 2012)

### Space-based mapping

**Figure 4.**
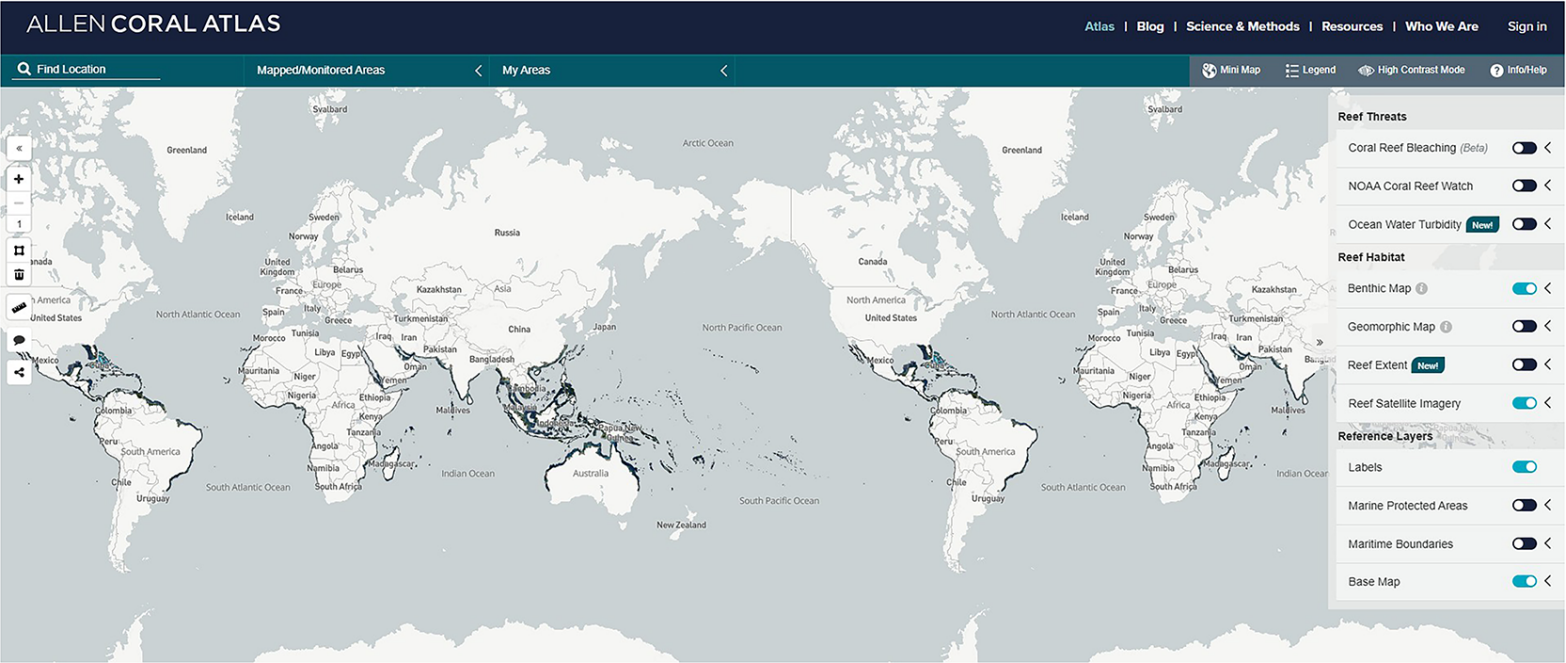
Distribution of tropical coral reefs. Screenshot from Allen Coral Atlas (2022) portal ( Mitchell *et al.*, 2022) Allen Coral Atlas | Atlas with more recent additions provided in ( Lyons *et al.*, 2024).

Space-based mapping platforms (i.e. Spaceborne platforms) allow one to map shallow habitats (< 20 m) at large spatial scales 10–100s of km ^2^, but often at low to coarse resolution (m ^2^- 100s km ^2^ scale). Spaceborne platforms consist of a variety of satellites with sensors able to provide satellite-derived bathymetry and satellite altimetry ( Selgrath *et al.*, 2016). For instance, satellite imagery represents a powerful tool to map the extent of shallow tropical coral reefs, data which has been used to create the Allen Coral Atlas ( Figure 4) ( Lyons *et al.*, 2024). In the review conducted by Nguyen *et al.* (2021) satellites, as a tool to map tropical coral reefs, are used in almost 50% of the studies and are recommended by the Coral Reef Expert Group for MAF mapping and change detection on a broad scale ( González-Rivero *et al.*, 2020). The popularity of using satellite data has increased, especially in recent years. E.g. in 2021, twice as many papers were published in the two years prior compared to in the last ten years ( Nguyen *et al.*, 2021). Satellite imagery is especially useful for mapping large spatial areas, such as at a global and regional scale, and for monitoring changes in habitat coverage over long time periods at an affordable price. For example, Palandro *et al.* (2003) demonstrated, by using satellite data, a loss in coral cover in the Carysfort Reef in Florida (USA), going from 52% in 1981 to 6% in 2000. Satellite imagery has also been used to map litter associated with tropical coral reefs and track the movement of megafauna, such as turtles and sharks, between coral reef areas.

However, there are a couple of aspects to consider when choosing satellite images. First, depending on using data from multispectral and hyperspectral satellites, the spatial resolution (i.e. the surface represented by a pixel) of the habitat one can map will be higher or lower. Currently, the best achievable resolution of hyperspectral sensors ranges from several tens of meters to up to 1 km ( Hedley *et al.*, 2016). In contrast, most multispectral sensors have a resolution better than 4 m. However, the resolution of satellites is quickly improving, and with it, the accuracy of coral maps. Secondly, the ability to use satellite data to map MAF also depends on sunlight penetration. Areas that have a lot of cloud cover may have clouds “blocking” the view, and light attenuation in water due to absorption (e.g. when there is higher turbidity or at deeper depth) and scattering (e.g. due to wave action) results in satellites often being unable to map accurately and/or below 20 m depth. While this approach has been shown useful for shallow shellfish beds ( Nieuwhof *et al.*, 2015), tropical coral reefs can extend beyond 20 m depth, and therefore, maps produced with satellite imagery may underestimate the true extent of habitats such as coral reefs that grow at deeper depths ( Eyal *et al.*, 2021; Galbraith *et al.*, 2022; Liaño-Carrera *et al.*, 2019).

### Air-based mapping

Airborne platforms consist of manned aeroplanes, helicopters and drones and unmanned aerial systems (UAS), which are used to map the seafloor at local and regional scales (1-to-100 km ^2^), and are most useful for mapping shallow MAFs (<20 m). Like satellites, airborne tools are also used to map tropical coral reefs ( Collin *et al.*, 2018; Hamylton, 2017). These platforms mostly use electromagnetic waves and primarily encompass aerial photography, active light detection and ranging (LiDAR) and radar imaging ( Javan *et al.*, 2025 and references therein). Depending on the closeness of the airborne vehicle to the ocean’s surface, a spatial resolution from several centimetres up to hundreds of metres can be achieved. Another advantage of using airborne mapping tools is that one can control the time of day and the flight direction, which can avoid issues with sunlight reflection and times with cloud coverage. They can also cover a relatively large area in a short time and can usually be operated for several hours per day. While drones are a relatively cost-efficient way to map an area, manned airplanes and helicopters are more costly.

**Figure 5.**
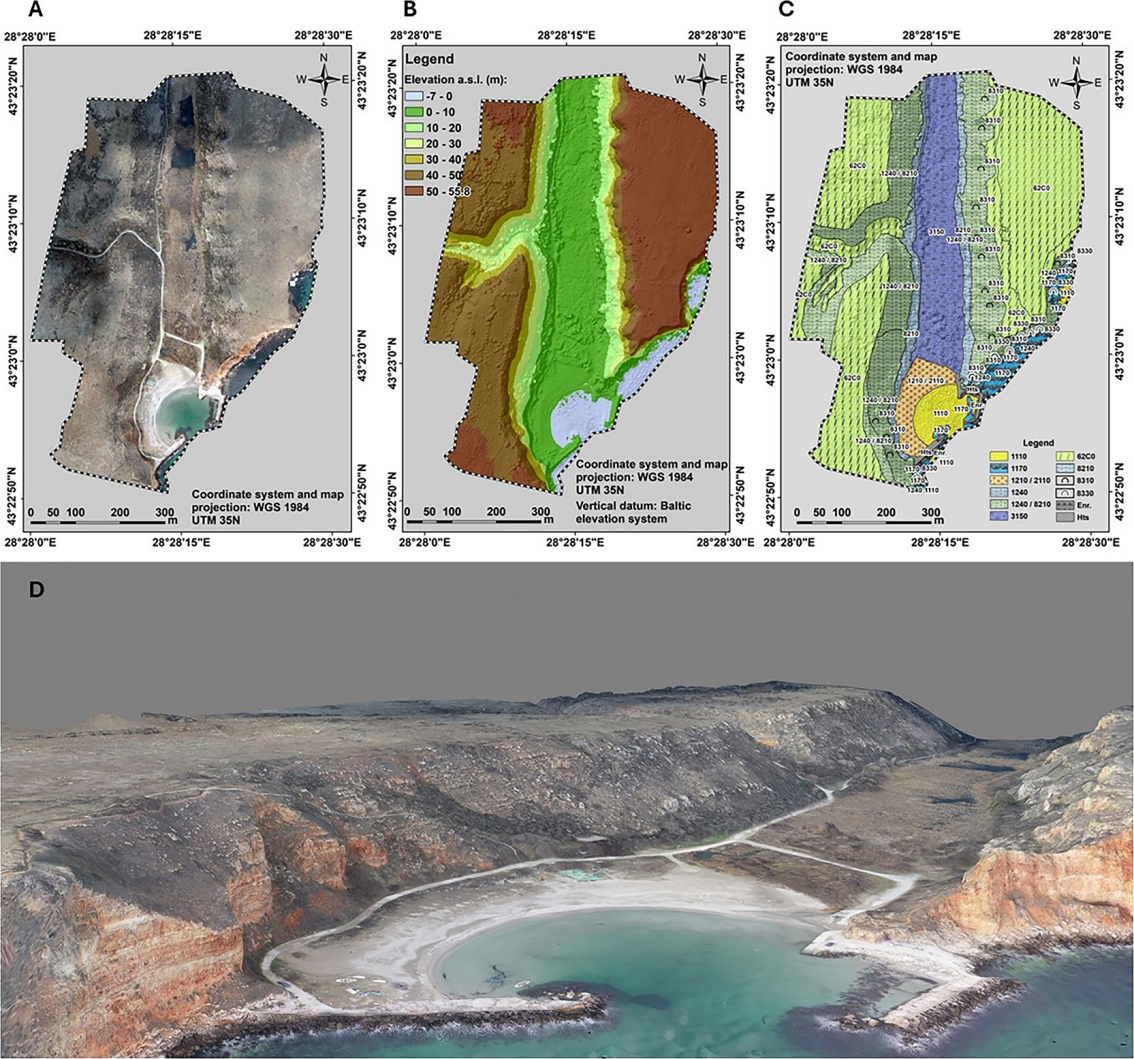
UAS-derived imagery products for MAF mapping purposes. Three of the basic drone products are an ( **A**) orthophotomosaic, ( **D**) 3D photorealistic model of the area, ( **B**) a (topo-bathy) DSM, all three at cm scale. ( **C**) The biotope/habitat map is the final stage of the analysis. The codes readable on the map provided are the HD habitat codes, i.e. 1110 - sandbanks, 1170 - reefs, 1210/2110 - Annual vegetation of drift lines/ embryonic shifting dunes, 1240 - Vegetated sea cliffs of the Mediterranean coasts with endemic Limonium spp, 8210 - Calcareous rocky slopes with chasmophytic vegetation, 3150 - Natural eutrophic lakes with Magnopotamion or Hydrocharition, 62C0 - Ponto-Sarmatic steppes, 8310 - Caves not open to the public, 8310 - submerged or partially submerged sea caves, Enr - enrockment (rip-rap), Hts - hydrotechnical structure/coastal engineering structure.

Systems such as UAS leverage aerial photogrammetry to generate high-resolution spatial data, contributing significantly to marine habitat monitoring and conservation efforts ( Figure 5) ( Monteiro *et al.*, 2021; Ventura *et al.*, 2023). By integrating Red Green Blue (RGB) and multispectral imagery, UAS-based MAF mapping enables the generation of digital surface models (DSM), 3-dimensional (3D) mesh models and RGB and multispectral orthomosaics ( Kvile *et al.*, 2024; Mazza *et al.*, 2023) ( Figure 5). Ground control points (GCPs) and georeferencing techniques enhance the spatial accuracy of the derived products, while bathymetric corrections improve mapping capabilities for submerged structures ( Alevizos *et al.*, 2022a; David *et al.*, 2021; Mandlburger *et al.*, 2021).

One of the main advantages of UAS technology is its ability to provide high-resolution data compared to satellite or aircraft-based remote sensing. Additionally, UAS surveys are cost-effective and offer a flexible alternative to traditional hydrographic methods ( Alevizos *et al.*, 2022a; Kvile *et al.*, 2024), allowing for rapid data collection across extensive areas with reduced time and resources. These advantages make UAS an indispensable tool for habitat classification, supporting machine-learning models in distinguishing different habitat types ( Giles *et al.*, 2023; Nababan *et al.*, 2021; Tallam *et al.*, 2023; Ventura *et al.*, 2023) ( Figure 5C). Furthermore, the generation of DSMs ( Figure 5B) and mesh models enables detailed morphological assessments of underwater features ( David *et al.*, 2021; Prodanov *et al.*, 2020), including shallow-water archaeological sites ( Mazza *et al.*, 2023; Prahov *et al.*, 2020), while multitemporal surveys facilitate change detection, helping monitor habitat changes due to both natural and anthropogenic factors ( Kvile *et al.*, 2024; Monteiro *et al.*, 2021).

Despite its many benefits, air-based mapping also faces several challenges. Water clarity and depth significantly impact the effectiveness of photogrammetry, as optical penetration is limited in deeper or turbid waters ( Alevizos *et al.*, 2022a; Mandlburger *et al.*, 2021). Environmental conditions such as surface reflections, wave motion, and lighting variations can also affect image quality and data accuracy ( David *et al.*, 2021; Kvile *et al.*, 2024). Moreover, regulatory constraints must be considered, as for example, UAV operations in coastal areas require compliance with aviation and marine protection regulations. Addressing these limitations requires the integration of advanced sensor technologies, improved image processing algorithms, and optimised flight planning strategies. Similarly to satellite-based images, air-based methods can only be used to map relatively shallow systems (~20 m depth). While repeated surveys are possible with this approach, often data will only be collected for the duration of a project (<10 years). It was only in the early 2000s that researchers started using drones to map and monitor wildlife. Therefore, it is unlikely that baseline data from before then is available for comparison. Consequently, most studies using airborne mapping approaches use the data to describe the extent, composition and complexity of the habitat.

### Sea-based mapping tools

***Active acoustic tools.*** Active acoustic sonar-based technologies emit sound pulses from a transmitter, which then travel to the bottom and, in some cases, penetrate the seafloor down to variable depths while reflecting the upwards part of the emitted energy, which is recorded by a receiver. This signal is then processed in order to obtain a graphical expression of the surface or subseafloor ( Dowdeswell, 2016; Jakobsson *et al.*, 2016).

***Side Scan Sonar profiling.*** Side Scan Sonar (SSS) has become an essential tool for underwater MAF mapping due to its ability to provide high-resolution acoustic imagery of the seafloor. By analysing differences in acoustic reflectance, researchers can infer sediment composition, habitat structures, and biological distributions. The effectiveness of SSS in MAF mapping lies in its capability to cover large areas efficiently while maintaining high spatial resolution. This combination makes it a useful data source which can be used in ecological analysis, to e.g. understand how the presence of MAF affects fish habitat use. (e.g. Blondel, 2009; Degraer *et al.*, 2008; Greene *et al.*, 2018; Klaucke, 2017; Sun *et al.*, 2021).

Despite its proven capability for high-resolution seabed imaging, Side-Scan Sonar (SSS) exhibits several critical limitations that must be addressed for rigorous data interpretation. Primarily, the method is designed for acoustic backscatter mapping rather than full bathymetric profiling, thus providing limited inherent three-dimensional depth information and functioning as a supplement, rather than a substitute, for systems like Multibeam Echosounders ( Shang *et al.*, 2019; Zhao *et al.*, 2018). Its data products often suffer from low positional accuracy unless co-registered with other systems ( Shang *et al.*, 2019). A key challenge is the high sensitivity to environmental noise (e.g., water-column turbulence and vessel motion) and the effect of acoustic shadows, which are generated by irregular topography (boulders or biogenic structures) and can simultaneously obscure large areas of the seabed, creating blind zones and potential misinterpretation of morphology ( Grządziel, 2023). Furthermore, the effective spatial resolution is not constant, as increasing survey speed or swath range to maximize coverage inherently reduces along-track resolution, representing a known trade-off ( Grządziel, 2023). This issue is compounded in complex environments, where SSS may underestimate small-scale structural complexity (such as individual boulders) and struggle to delineate clear habitat boundaries in heterogeneous habitats ( Smith *et al.*, 2023; von Rönn *et al.*, 2019). Finally, the interpretation of acoustic mosaics remains operator-dependent and can be highly subjective without rigorous calibration and mandatory ground-truth validation, underscoring the necessity of standardisation for cross-survey comparability ( Blondel, 2009; Helferty & Johnson, 2019).

SSS operates by transmitting acoustic pulses from a transducer mounted on a towed vehicle, an autonomous underwater vehicle (AUV), or a ship. These pulses propagate through the water column and reflect off the seafloor, with the returning signals recorded to generate an acoustic image. The intensity of the returned signal, or backscatter, varies depending on the texture, hardness, and composition of the seabed ( Ainslie, 2010; Blondel, 2009). Backscatter measurements, often used in combination with bathymetric data, provide insights into the distribution of different habitat types. Areas with high backscatter typically correspond to hard substrates such as rocky outcrops, whereas low backscatter is associated with soft sediments like mud and sand ( Lurton *et al.*, 2015).

**Figure 6.**
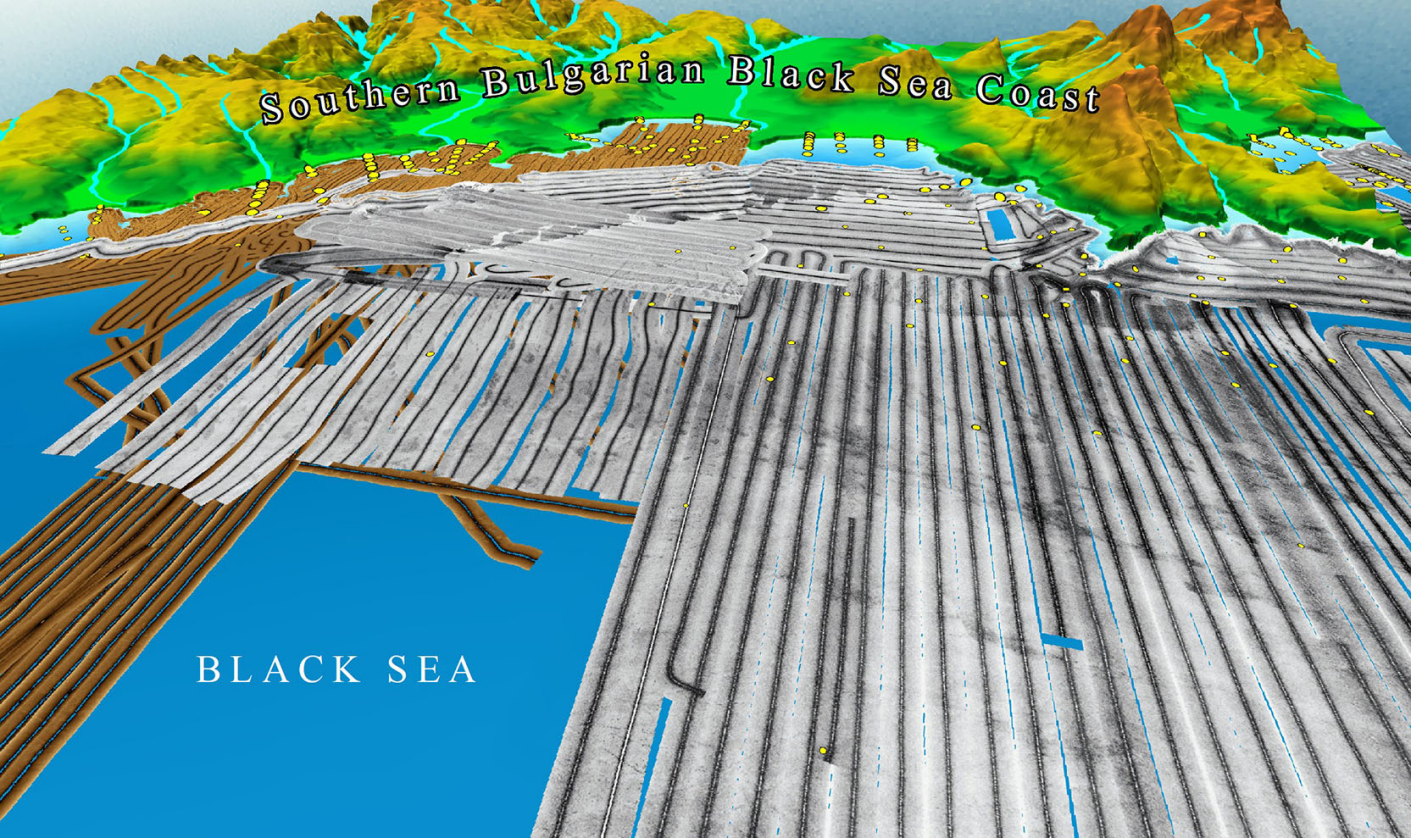
Side-scan sonar mosaics from the "Ropotamo" MPA, Southern Bulgarian Black Sea Coast ( Dimitrov *et al.*, 2019; Todorova *et al.*, 2015).

The data acquired from SSS surveys is processed into various products that aid in habitat classification and environmental assessments. These include (a) sonograms, which are raw acoustic images that display variations in backscatter intensity, used to visually interpret habitat types and seabed structures; (b) mosaics, which are composite images created by stitching together multiple sonar passes to form a continuous, large-scale representation of the surveyed area ( Figure 6); (c) backscatter maps, which are georeferenced representations of sonar intensity values, aiding in quantitative analysis of seabed properties; (d) habitat classification maps, which are interpreted maps where seabed types and biological zones are categorized based on sonar data and ground-truth samples; (e) three-dimensional models, which, when integrated with multibeam bathymetry data, allow the development of 3D representations of underwater landscapes, enhancing the visualisation and analysis of geomorphological features, substrate heterogeneity, and habitat complexity. These models improve our understanding of how seafloor morphology influences species distribution, sediment transport, and habitat connectivity, providing a powerful tool for ecological and geological interpretation (e.g., Blondel, 2009; Canals *et al.*, 2020; De Clippele *et al.*, 2017; Dimitrov *et al.*, 2019; Pillay *et al.*, 2020).

One of the primary applications of SSS in MAF mapping is classifying benthic environments based on substrate type and structure. This is critical for MAF mapping because the strength of the sonar signal provides crucial information about the composition of the seafloor, which is a key determinant of MAF distribution, as many sessile organisms like corals and sponges require stable, hard substrates for attachment, resulting in high-backscatter intensity areas. For example, studies have correlated high-resolution SSS images with sediment grab samples to classify seabed habitats in fisheries management and the resulting acoustic imagery is highly effective at capturing the texture and roughness (rugosity) created by biological structures like the biogenic reefs of tube-worms or bivalves, allowing for the efficient delineation of areas with rock or coarse sediment required by certain MAF species ( Lucieer, 2008; Micallef *et al.*, 2012; Papatheodorou *et al.*, 2012; Todorova *et al.*, 2015).

Manual interpretation of SSS imagery can be time-consuming and subjective. Therefore, automated image classification techniques have been developed to improve accuracy and efficiency. These techniques are increasingly vital for MAF research as the vast spatial extent of habitats like deep-sea sponge grounds or patchy cold-water coral reefs often results in massive datasets. Algorithms and supervised classification methods have been used to distinguish between different habitat types, achieving classification accuracies above 80% ( Brown & Collier, 2008; Kaeser *et al.*, 2013; Ojeda *et al.*, 2004; Rusmadi & Hasan, 2020; Sun *et al.*, 2020) and accelerating the creation of full-coverage MAF distribution maps, reducing the subjectivity associated with manually interpreting the subtle changes in backscatter that indicate biological structures.

SSS is extensively used to study the spatial extent, geomorphology, and geological composition of underwater landscapes, including reef structures, sedimentary deposits, and tectonic features. By integrating SSS data with ground-truthing methods such as sediment coring, bottom photography, and high-resolution bathymetry, researchers can characterise geomorphic features such as underwater ridges, sandbanks, rock formations, and fault structures. For example, high-resolution SSS mosaics have been instrumental in identifying lithological variations, fault structures, and sediment transport patterns, contributing to improved geological and geomorphological mapping of continental shelves and reef ecosystems ( Blondel, 2009; Dimitrov *et al.*, 2019; Pillay *et al.*, 2020; Subarsyah & Arifin, 2019; Todorova *et al.*, 2015; Wang *et al.*, 2023; Zhao *et al.*, 2018).

Advancements in sonar technology and image processing are set to transform MAF mapping with SSS. Machine learning models for automated classification and real-time data processing will enhance mapping accuracy, reducing subjectivity and increasing efficiency ( Pijanowski & Brown, 2021). The integration of SSS with AUVs and ROVs can facilitate high-resolution, real-time habitat assessments, expanding sonar applications in deep-sea exploration and environmental monitoring ( Wynn *et al.*, 2014). Additionally, emerging multi-frequency and multispectral sonar systems improve substrate discrimination, offering more detailed seafloor characterisation ( Gaida *et al.*, 2018). The combination of SSS with other remote sensing technologies, such as LiDAR and satellite-derived bathymetry, will further refine seabed classification and MAF mapping ( Ierodiaconou *et al.*, 2018).

Despite certain limitations, SSS remains a cornerstone of underwater MAF mapping, providing high-resolution acoustic imagery essential for marine science and conservation. Global initiatives like Seabed 2030 leverage advancements in sonar to map the entire ocean floor at high resolution, addressing knowledge gaps in marine ecosystems ( Mayer *et al.*, 2018). By overcoming current challenges and harnessing new technologies, SSS will continue to play a key role in sustainable ocean management, supporting biodiversity protection and ecosystem monitoring.

***Seismic reflection profiling.*** Seismic reflection profiling is a widely used tool to image the subseafloor at shallow (1–10’s m ^2^), intermediate (10’s to a few 100’s m ^2^) and large depths (several 100’s of meters to kilometres scale) ( Crutchley & Kopp, 2018). This method can be used to reveal information on the formation and history of certain MAFs, as best illustrated by cold-water coral carbonate mounds, which encompass a large variety of organisms other than corals.

The method relies on the emission of sound pulses to obtain a graphical expression of the subseafloor structure in the form of a seismic reflection profile ( Jakobsson *et al.*, 2016). Seismic reflection profiling requires an energy source, a receiving system of the reflected energy, a recording unit for data storage, and processing and visualisation software at various stages (e.g. pre-processing or post-processing). Acoustic impedance contrasts are stronger at interphases separating materials with distinct physical properties, such as sediments from underlying rocks, or sedimentary layers of different densities. These interphases are known as “seismic reflectors”. Consequently, the continuous repetition of emission-reception cycles over time allows for visualising the configuration of materials in the sub-seafloor along the time axis, including the boundaries between different geological units and their internal structure. That is why seismic reflection profiles usually have a vertical axis in two-way travel time (in seconds or milliseconds), expressing the time required for the signal to travel from the location of the source (at the sea surface or below) to the different reflectors at depth and back. The horizontal axis is also expressed in time, which in this case represents the time required for successive emission-reception cycles while the vessel moves forward at a controlled speed ( Jakobsson *et al.*, 2016).

There is a wide variety of seismic reflection systems, each designed for a specific purpose. The most interesting for MAFs and other shallow subseafloor studies are very high to high-frequency systems, which allow for penetrating the upper sediment layers while providing centimetre to decimetre-scale resolutions. Other high-resolution towed seismic reflection systems, such as boomers or small sparkers, could also be of interest, although they are intended to achieve larger depths below the seafloor than those strictly needed for MAFS studies. In these systems, both the source (emitter) and the receiver system are towed behind the mother vessel. Receivers usually consist of a flexible pipe (named streamer) holding a set of hydrophones to detect the reflected acoustic signals. Acquisition can be single-channel or multi-channel, and 2D or 3D (seismic cubes). Grids of densely spaced 2D profiles can be used to produce pseudo-3D seismic cubes. For example, sub-bottom profiler data have been used, in combination with side scan sonar data, for benthic MAF mapping in a number of places worldwide (e.g. Alevizos *et al.*, 2022b; Fakiris *et al.*, 2018; Hamouda *et al.*, 2024; Ierodiaconou *et al.*, 2018; James *et al.*, 2007).

***Multibeam sonar.*** Since the early 1990s, multibeam echosounders have become the dominant method for full-coverage seafloor mapping at all depths ( Menandro & Bastos, 2020). Similar to seismic reflection systems, multibeam systems require the emission of repeated energy pulses at high frequencies that travel and are reflected off the seabed, returning to the surface or the location of the survey vehicle. Today, there is a wide variety of multibeam systems on the market, some of which are designed for shallow coastal waters and small-sized boats or underwater vehicles, whereas others are intended for ocean depths and can only be installed in large vessels. The diversity of multibeam systems can be seen on the webpages of the leading manufacturers, such as Atlas Hydrographic, Konsberg Discovery, LC Klein, Norbit, Teledyne RESON A/S, or Wärtsilä Elac Nautik.

As indicated by their name, multibeam systems consist of arrays of emitters and receivers, each of which emits a beam with specific properties (e.g., amplitude, single-beam opening angle). Adjacent beams form a fan-like front that successively insonifies seafloor stretches of a given total width as the carrying platform advances, which can be either a surface vessel or an underwater vehicle. Successive swaths expanding towards both sides of the platform provide full coverage of the insonified area along a given stretch of the seafloor. The width of the mapped seabed stretch depends on water depth (i.e., the larger the water depth, the wider the stretch) and on system configuration (i.e., the larger the total opening angle of the beam ensemble, the wider the stretch) ( Jakobsson *et al.*, 2016). Overlapping successive, generally parallel, stretches allow for achieving full coverage bathymetric maps of given regions, which are essential for MAF mapping and understanding, including MAFs. Further, any kind of information, such as landform features or species presence, could be draped on the bathymetry base map using geospatial software such as ArcPro or QGIS (e.g. Sowers *et al.*, 2024)

The footprint of every individual beam determines the resolution of the bathymetric products obtained from multibeam systems, such that smaller footprints (involving less energy and higher frequencies) result in higher-resolution maps. However, to travel deeper, more energy is needed, which usually implies that deep-water bathymetric maps have poorer resolution than shallow water ones, unless using an underwater vehicle capable of carrying the system close to the seabed. Vessel speed while profiling must be as constant as possible and not too high (i.e., usually around 8 knots) to ensure the quality of the data, though this ultimately depends on vessel’s performance and sea state. Usually, multibeam systems are hull-mounted, although they can also be attached to ROVs and AUVs, resulting in very high-resolution data. For example, at the Mingulay reef complex a “microbathymetry” was collected through attaching a MBES to an ROV. This resulted in a map of a 20 cm resolution on which live cold-water coral colonies were visible and annotated for use in a species distribution model ( De Clippele *et al.*, 2017).

Multibeam data enable the creation of a variety of mapping products that can be particularly useful. These include backscatter intensity maps, which represent the intensity of ”backscattered” seabed reflection of the emitted signal, itself indicative of the seabed’s nature, either rocky or sedimentary or vegetation-covered. Another option is generating 3D Digital Terrain Models from full coverage bathymetry datasets, allowing visualisations from different angles and elevations, with more or less vertical exaggeration of seascape images to better visualise specific features ( Fabri *et al.*, 2017). Other cartographic products directly derived from multibeam bathymetry data, which are increasingly being used, include rugosity (a dimensionless measure of surface roughness), slope gradient, bathymetric positioning index, and aspect (slope orientation), and segmentation (per backscatter classes, for instance) maps ( Canals *et al.*, 2020; De Clippele *et al.*, 2017 and references therein).

If repeated at given intervals of time, such full-coverage maps allow for the monitoring of seascape and habitat evolution in an unprecedented way. Repeated multibeam surveys have been done to assess both short and long-term changes associated with the seafloor (e.g. Normandeau *et al.*, 2020). The “Digital Elevation Model (DEM) of Difference” (DoD) can be calculated and will indicate the change in elevation with positive values showing deposition (or fill), negative values showing erosion (or cut, scour) and null values showing an unchanged surface ( Schmitt *et al.*, 2008). However, the accuracy of the depth values can be affected due to different sonar systems being used, vessel-related differences because of configuration and weather conditions, differences in oceanographic conditions and issues related to the use of geo-positioning techniques ( Brothers *et al.*, 2011; Schmitt *et al.*, 2008). These inaccuracies may be more pronounced in deeper locations and areas with more complex hydrodynamics, such as cold-water coral reefs. Given enough time and under the right environmental conditions, cold-water coral reefs can develop from mini-mounds to large, cold-water carbonate mounds. While these mounds can be mapped and characterised using multibeam sonar approaches, to date no studies have assessed long-term changes in their morphology. Using multiple acoustic frequencies could also be useful as it may help differentiate seafloor features of various sizes and shapes ( Whitmire *et al.*, 2007).

***Cameras.*** Image-based tools, such as cameras that capture still images or video, are widely used in MAF mapping. These systems typically provide centimetre-scale resolution and cover relatively small spatial areas (usually less than 1 km ^2^). Cameras can be operated by divers, deployed from vessels using various support systems (e.g., drop or drift cameras), or mounted on underwater vehicles such as ROVs and AUVs ( De Clippele *et al.*, 2017; Foubert *et al.*, 2011; Huvenne *et al.*, 2011; Robert *et al.*, 2017; Van Audenhaege *et al.*, 2021).

The characteristics of the vehicles and camera systems, particularly in terms of stability, lighting, and control, influence the type and quality of data collected, making each platform more suitable for specific objectives or environments.

ROVs are controlled from a surface vessel, allowing real-time decision-making and fine manoeuvrability. This makes them ideal for targeted observations, detailed inspection of specific areas, or sample collection. Their lighting systems can be adjusted on the spot to adapt to local visibility conditions. However, their dependence on a ship-based control system and umbilical cable limits the area they can cover and may introduce motion constraints.

AUVs, on the other hand, are autonomous platforms that follow pre-programmed missions without human control during deployment. They are especially well-suited for high-resolution mapping of small to medium-sized areas due to their stable motion and efficient, systematic coverage. AUVs often carry advanced cameras and lighting systems calibrated for optimal imaging conditions. While they offer high spatial resolution and consistency, they lack flexibility during missions and cannot adjust to unexpected features in real time.

High-resolution (<1 cm) video mosaics, combined with multibeam sonar data, typically mounted on AUVs, have provided valuable insights into the spatial organisation of deep-sea coral frameworks and the environmental and temporal drivers influencing them ( Boolukos *et al.*, 2019; Conti *et al.*, 2019; Price *et al.*, 2019). As a result, non-invasive methods involving remote imaging technologies are increasingly favoured for studying and monitoring marine benthic habitats.

The quality of the imagery depends on several factors, including the camera specifications, lighting conditions, and water turbidity. Image resolution is generally highest when the platform is stationary or moving slowly. Collecting data close to the seafloor improves the ability to identify organisms and benthic structures associated with MAFs. However, operating very close to the bottom may pose safety concerns and increase the risk of disturbing or damaging fragile marine life.

### Sea-based platforms

There are three categories of sea-based mapping platforms: i.e. those at the surface, underwater and those held by divers and snorkellers. Sea surface platforms comprise vessels of different sizes and capabilities, including Unmanned Surface Vehicles (USV). Underwater platforms comprise manned submersibles, unmanned tethered Remotely Operated Vehicles (ROV) and untethered Autonomous Underwater Vehicles (AUVs). Divers could be considered “living underwater platforms”. Active acoustic (e.g. seismic and multibeam sonar) and image-based tools are attached to sea-based mapping platforms to collect depth or backscatter information of habitats at local and regional scales. Beyond very shallow coastal waters, a combination of sea surface platforms for medium to large-scale general mapping (i.e. from km2 to 100’s of km2 in size areas) with underwater platforms for detailed inspection and mapping of specific locations, previously identified in the general maps, is a commonly used approach when mapping MAFs.

The advantage of surface-based mapping platforms, such as ships, is that they can travel at greater speed and cover larger areas. It is relatively easy to follow a straight trajectory and maintain a constant speed (i.e., usually 5–8 knots). Fair sea conditions are also needed to collect high-quality data. Both single-beam and, especially, multibeam data have been used to produce seafloor maps at various scales, from local (e.g. De Clippele *et al.*, 2017) to basin-scale (e.g. Schumacher *et al.*, 2022). One disadvantage when working with multibeam systems is that in deeper habitats, the resolution of the maps is lower due to the larger distance between the seafloor and the hull of the surface-based vessel.

Whereas there are some manned submersibles in use across the world, the number of unmanned vehicles largely outweighs the manned ones, mostly because of safety reasons and associated operational procedures and costs. The advantages of underwater platforms are that their design can be changed depending on each specific mission's needs and that they can operate at very short distances from the seafloor, thus enhancing data resolution. An inconvenience of these platforms is that they move at relatively low speeds, can struggle to hold position and can usually operate for only several hours a day, although longer continuous periods (e.g. several days-weeks) could be envisaged in the case of AUVs. Longer deployment times could allow for covering larger or more remote areas, or could allow for repeat surveys to understand changes in diel patterns.

Divers can carry a variety of instruments (e.g., video and photographic cameras), and assist in detailed mapping and, more importantly, in the in-situ ground truthing of data acquired from other platforms. For example, they can check in situ if a given substrate is hard or soft, a property that is often not obvious from imagery alone. However, the disadvantage if using divers is that they can only reach ~30 m water depth and that they can only spend a limited time underwater (~45–60 min, but 4–8 hours with a rebreather). Consequently, the area they can cover during surveys can be limited.

## Analysis approaches

**Figure 7.**
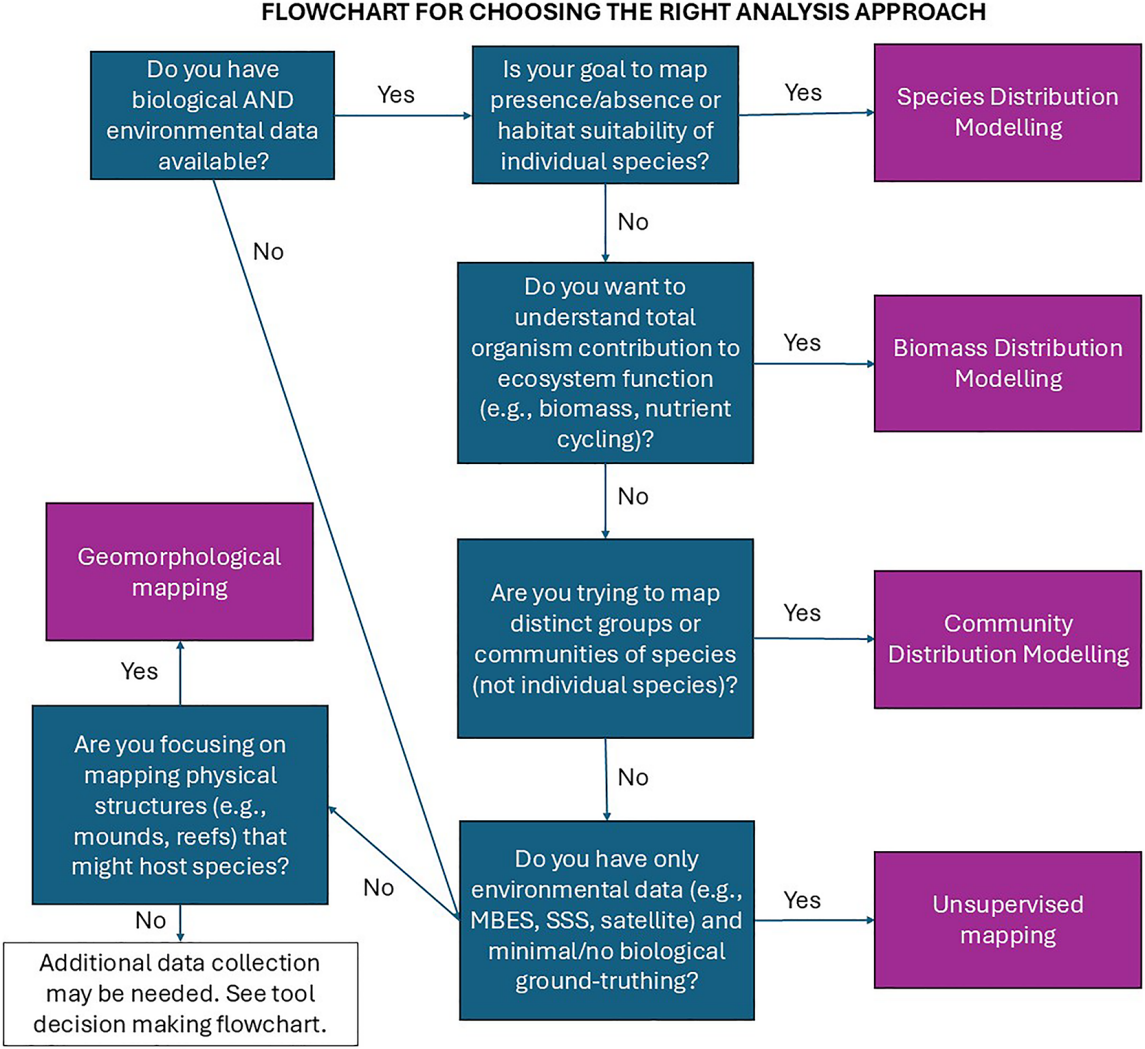
Flowchart to help a user decide which analysis approach to use.

Supervised and unsupervised mapping and modelling approaches are being used to either automate MAF mapping or characterisation, or to predict habitat or species presence for areas where biological data is missing but environmental data is available ( Figure 7). These methods provide a more detailed understanding of the environmental envelope in which species and habitats occur, and can therefore be used to predict how the distribution of habitats and species may change under different climate change scenarios (e.g. Morato *et al.*, 2020; Schumacher *et al.*, 2022; Vinha *et al.*, 2025). Two types of data are required to produce a model: biological (response) data and environmental (predictor) data.

Biological data usually comes in the form of image data or physical samples. Thanks to global open-access initiatives, a substantial amount of biological data is now available through open-source platforms such as the Global Biodiversity Information Facility (GBIF; https://www.gbif.org/) or the Ocean Biodiversity Information System (OBIS; https://obis.org/) and researchers are publishing their datasets on online data repositories (e.g., PANGAEA, Dryad or Figshare). GBIF functions as a global repository for species occurrence records, aggregating data from diverse sources including museums, research institutions, environmental monitoring programs, and citizen science initiatives. OBIS, developed under the auspices of the Intergovernmental Oceanographic Commission of UNESCO, provides georeferenced data on the distribution of marine species collected through research cruises, monitoring programs, and other marine surveys. Although OBIS has large data gaps, with a focus on data collected in western areas, it aims for global coverage. Together, these platforms significantly enhance the accessibility and integration of marine biodiversity data.

Among the most common environmental parameters, critical for the spatial distribution of the MAF species are bathymetry and morphological parameters describing 3D microhabitats, the substrate, the direction and intensity of marine currents, the roughness of the sea bottom and its orientation, temperature, salinity and nutrients concentrations (e.g. Chimienti *et al.*, 2019; Chimienti *et al.*, 2020). Terrain variables, such as roughness, orientation, depth, bathymetric positioning index and slope, can be calculated from bathymetry data in R or GIS (e.g. De Clippele *et al.*, 2017; Ramiro-Sánchez *et al.*, 2019). The direction and intensity of marine currents can be measured with current speed sensors and acoustic doppler current profilers (ADCPs). Temperature, salinity and depth can be measured with Conductivity, Temperature and Depth (CTD) sensors. Finally, nutrient concentrations can be measured with sediment traps, Chl-a sensors or even satellite data (e.g. Vad *et al.*, 2025). Information on the substrate can be either extracted from images/videos, from backscatter data or from physical samples. Much relevant data is now also available as part of open-access databases, such as GEBCO bathymetry ( Mayer *et al.*, 2018) (providing a global bathymetric grid). Copernicus Marine Service (providing different physical and chemical oceanographical data products), Bio-Oracle ( Assis *et al.*, 2018; Assis *et al.*, 2024; Tyberghein *et al.*, 2012) (providing current and future physical and chemical oceanographic datasets), NASA Earthdata ( https://www.earthdata.nasa.gov/) and or European Space Agency Climate Office ( https://climate.esa.int) (providing satellite imagery of environmental conditions.

### Unsupervised MAF mapping

Unsupervised underwater MAF mapping is a technique that utilises algorithms to map elements with segmented data, followed by the assignment of biological information, such as habitat type, identified from ground-truth observations made by a camera or physical sampling (e.g., Calvert *et al.*, 2015; Lamarche *et al.*, 2016; Schumacher *et al.*, 2022). This approach is most effective in marine environments where sampling data may be scarce. It has traditionally been the most common method of conducting benthic mapping ( Misiuk & Brown, 2024). A significant data source for shallow waters comes from electromagnetic remote sensing technologies (e.g., laser scanning or LiDAR, and multispectral or hyperspectral cameras) providing images or products that can be processed using unsupervised methods to map underwater features, especially coral reefs ( Misiuk & Brown, 2024). However, acoustic data technologies, such as MBES or SSS, are among the most common sources of data for MAF mapping ( Brown *et al.*, 2011b), owing to their ability to provide full-coverage bathymetry and backscatter data from shallow to deep-sea environments.

The use of unsupervised models for processing environmental data has significantly increased over the past decade, replacing manual interpretation by experts. These models aim to identify meaningful patterns in environmental variables without relying on response information ( Calvert *et al.*, 2015). They include various machine learning and clustering algorithms such as k-means, Self-Organizing Maps (SOMs), DBSCAN and OPTICS (e.g., Fendereski *et al.*, 2014; Hoang *et al.*, 2016; Menandro *et al.*, 2022; Weslawski *et al.*, 2013). Another novelty unsupervised technique for benthic mapping is Simultaneous Localisation and Mapping (SLAM), which allows AUVs to simultaneously map their environment and determine their location within it using probabilistic and iterative methods ( Liu *et al.*, 2024). Also, Deep Learning Methods such as Autoencoders or Generative Adversarial Networks (GANs) are being used in assessing and mapping benthic marine habitats ( Burns *et al.*, 2022; Massot-Campos *et al.*, 2023; Yamada *et al.*, 2021).

In unsupervised habitat characterisation, clusters represent statistically similar groups of environmental or acoustic features that may correspond to distinct habitat types. These clusters are subsequently interpreted and validated using ground-truth data, most commonly underwater imagery, which provides wider spatial coverage than physical samples collected by drags or grabs ( Brown *et al.*, 2011a). Additionally, it is a non-invasive method, particularly suitable for complex areas such as rocky bottoms or slopes, without causing significant damage to the ecosystem.

### Geomorphological mapping

Certain MAFs, such as cold-water coral, bivalve and polychaete reefs either form elevated biogenic structures or grow on them. Therefore, mapping these structures can reveal information about their distribution. Such structures are reefs or mounds. Their presence can indicate information on the abundance of vulnerable marine habitats in an area ( Ramiro-Sánchez *et al.*, 2019), and their geomorphological characteristics can be used as proxies for environmental conditions as part of species distribution models ( De Clippele *et al.*, 2017). Manually delineating these features of interest can be very time consuming and can result in non-repeatable results ( Arosio *et al.*, 2024). Therefore, there have been several semi-automated approaches developed.

Gafeira *et al.* (2018) developed a GIS tool to map pockmarks, which was adapted to map coral mounds in De Clippele *et al.* (2017), and has recently been made available as an open-source ArcMap Toolbox, i.e. the CoMMa toolbox ( Arosio *et al.*, 2024). This tool is very flexible and allows one to map both elevated structures and depressions. It allows for the delineation and characterisation of features in just a few repeatable steps. Object-based approaches have also been used to automatically derive maps of coral carbonate mounds in Norway ( Diesing & Thorsnes, 2018).

### Species distribution modelling

Several algorithms are available for use in species distribution modelling (SDM) ( Barker & MacIsaac, 2022, table 2, Winship *et al.*, 2020); these are defined by the availability of presence data and their number, the occurrence of real absence data or the need for random creation of pseudoabsences. Several workflows have been developed in R ( Guéguen *et al.*, 2025; Kass *et al.*, 2023; Naimi & Araújo, 2016). Depending on e.g. the size of the dataset and the level of interpretability, or the accuracy of the model’s prediction needed, one may choose one approach over another. It is important for the user to understand the pros and cons of each modelling approach before applying or interpreting the results.

SDM outputs are spatially explicit maps that also include information on the response of the studied species to the used environmental data, referred to as “response curves”. The maps, as a spatial information can be used and is already, for spatial prioritisation modelling when new Marine Protected Areas (MPAs) are needed to be designed or the current designed are under review based on the Intergovernmental Panel on Climate Change (IPCC) climate change scenarios, under the SPPs, where adaptation to the current MPA boundaries have to be checked under various climate change scenarios ( Adam *et al.*, 2021; Doorga *et al.*, 2023; Principe *et al.*, 2021). Such a combined use of modelling is useful and paves the road for adaptive designation of MPAs in the future.

### Biomass distribution modelling

The extent of a species does not necessarily reveal the extent of its contribution to ecosystem processes, such as carbon and nitrogen turnover, in an area. This is because an organism’s size and its biomass can vary across environmental and temporal gradients, influencing the amount of nutrients being turned over ( Clark *et al.*, 2024; De Clippele *et al.*, 2021a; De Clippele *et al.*, 2021b; Greiffenhagen *et al.*, 2024).

Mapping biomass variations in MAFs is a complex task. In terrestrial ecosystems, large-scale biomass mapping is often supported by remote sensing technologies like satellite imagery, which are combined with field data ( Baccini *et al.*, 2008). While satellite imagery has been used to map coral reefs, its application to MAFs is hindered by the variation in depth and species diversity, which reduces its accuracy for biomass estimation. Any habitats deeper than 30 meters are likely to be missed by satellite imagery alone, making this approach unsuitable for deep-sea environments. Although periodic and continuous photographic surveys of the seafloor are becoming more common ( Boolukos *et al.*, 2019; Bodenmann *et al.*, 2023; Conti *et al.*, 2019; Van Audenhaege *et al.*, 2021; Van Audenhaege *et al.*, 2024), their large-spatial-scale application remains limited.

To address the challenge of accounting for spatial biomass variation and improve the accuracy of ecosystem-level nutrient turnover estimations, De Clippele *et al.* (2021a) developed a novel "biomass mapping approach." This method leverages machine learning models that integrate biological, environmental, and ecosystem function data to map the spatial variation of species within a given area. The biomass values derived from this mapping can then be used to calculate the overall nutrient turnover for the modelled species across the entire ecosystem ( De Clippele *et al.*, 2021a; De Clippele *et al.*, 2021b; Greiffenhagen *et al.*, 2024). The approach relies on surface area data extracted from images ( De Clippele *et al.*, 2021a). These measurements can be obtained through various methods: manually using Image J software ( De Clippele *et al.*, 2021a), semi-automatically using Photoshop ( De Clippele *et al.* 2021b; Greiffenhagen *et al.*, 2024), or could be done fully automatically ( Clark *et al.*, 2024).

### Community distribution modelling

**Figure 8.**
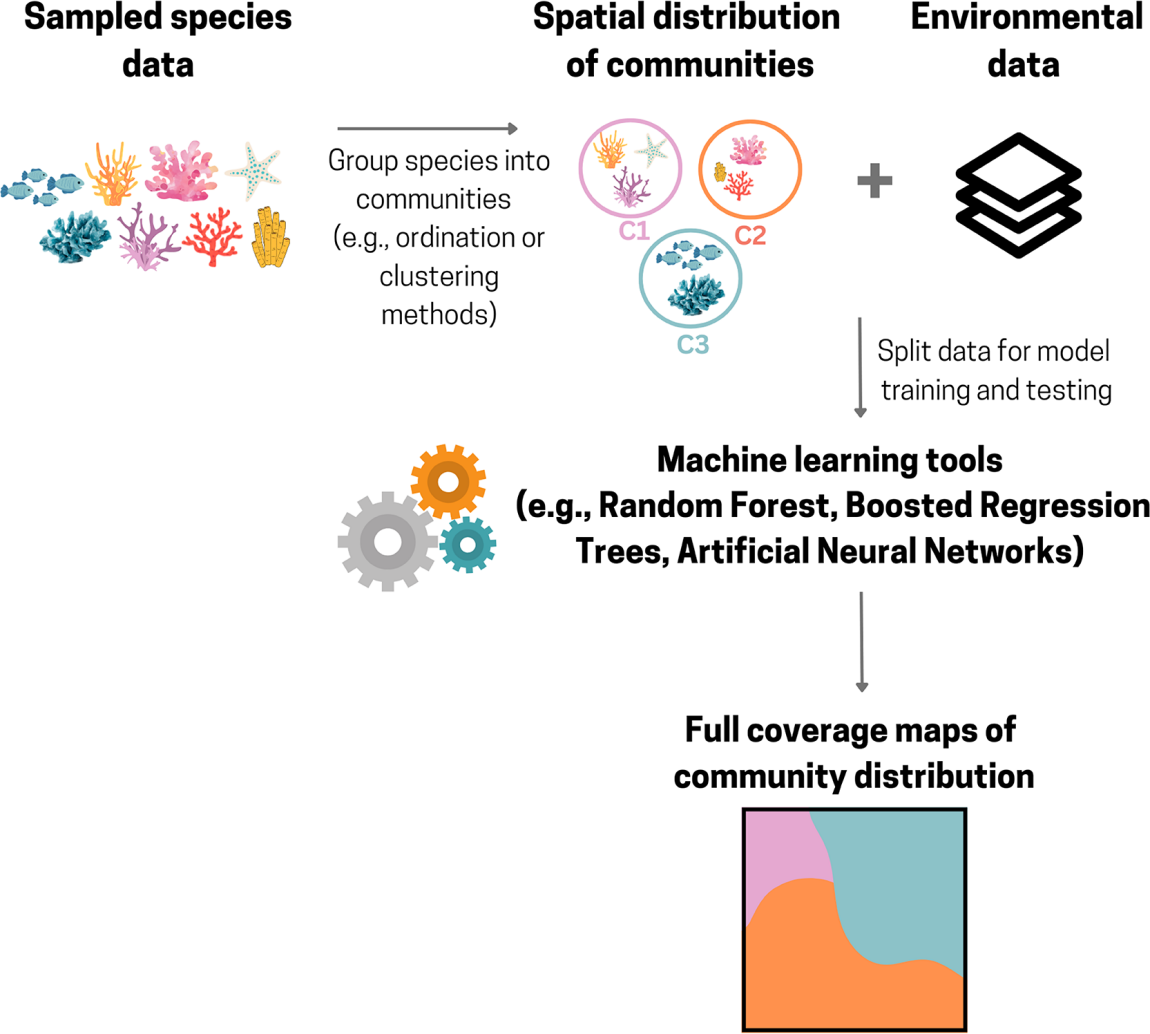
Schematic representation of a workflow that can be used to create predictive community maps of Marine Animal Forests. Created in Canva.

Instead of modelling the distribution of species individually, it is also possible to model the distribution of species assemblages or communities associated with MAFs (e.g. Vinha *et al.*, 2025). Community-based models can be developed using a similar approach to species distribution models (SDMs) (see previous section), where spatial distribution data of communities and relevant environmental variables are used to predict their distribution into novel areas. In these models, rather than using a dataset of presence-absence records for individual species (i.e. univariate model), a dataset with multiple categories representing different species assemblages or communities (e.g., community 1, community 2, community 3, etc.) (i.e. multivariate model), with their respective spatial distribution, is used. Machine learning techniques can be particularly well-suited for this type of modelling due to their ability to handle a multi-class response variable. For example, Random Forest classifications have been used to create full coverage maps of community distributions on seamounts ( Goode *et al.*, 2021; Vinha *et al.*, 2025) ( Figure 8), where environmental variables were used to predict the distribution of communities beyond sampled areas.

If spatial data on communities is not available, but instead, spatial data on different species in a study area is available, Joint species distribution models (JSDMs) might be a useful approach for community modelling ( Roberts *et al.*, 2022; Stephenson *et al.*, 2024). This method allows for predicting the distribution of multiple species simultaneously, being useful to evidence species interactions and co-occurrence patterns within a community ( Pollock *et al.*, 2014).

## Conclusion and future directions

Future developments in sensor integration, machine learning (ML) algorithms, AI-driven classification, and advanced photogrammetric processing will further enhance its capabilities in underwater MAF mapping to classify coastal habitats with minimal or no field data collection ( Kvile *et al*., 2024; Liu *et al*., 2022). However, both lower resolution and manual approaches remain having a key value, especially in unmapped or unexplored areas, which still comprise the majority of our oceans. Other novel approaches such as environmental DNA (eDNA) show promise for detecting the presence of species, and to study habitat-species interactions and may be used to map MAF in the future ( Bizzozzero *et al.*, 2025; Wilms *et al.*, 2022).

This overview of tools and approaches serves as a guide for users to assess which approach they may want to use in specific situations. Mapping the seafloor at various resolutions and through different methods is crucial for conservation efforts, as it enables a more comprehensive understanding of habitat distribution, biodiversity, and biomass variations, ultimately informing more effective and targeted management strategies for preserving marine ecosystems.

## Ethics and consent

Ethical approval and consent were not required.

## Data Availability

There is no data associated with this article.
