# Supplementary material for: Tools and approaches for mapping Marine Animal Forests: A practical overview for researchers and conservationists
Source: Open Res Eur. 2026 Feb 14;5:272. Originally published 2025 Sep 5. [Version 2] doi: 10.12688/openreseurope.20823.2 (PMC13000406; doi:10.12688/openreseurope.20823.2)
Supplement: Peer review history

# Reviewer report: Reviewer response for version 2

**DOI:** 10.21956/openreseurope.23645.r70543

**Published:** 2026-03-18

**Authors:** Khaira Ismail

**Affiliations:** 1 Universiti Malaysia Terengganu, Kuala Terengganu, Terengganu, Malaysia

1. Established methods are well-covered; applicability guidance for practitioners could be strengthened. The review focuses on commonly used, proven approaches - appropriate for a practical overview. However, practitioners would benefit from clearer guidance on:

 (a) Which MAF types are suitable for which tools? For example, the habitat mapping section discusses coral reefs extensively (a reasonable focus given their accessibility), but does not clarify how these approaches translate to sponge grounds, polychaete reefs, or bivalve reefs.

 (b) When are certain tools unsuitable? For instance, the seabed platforms subsection discusses methods in general without clarifying depth limitations, water clarity requirements, or which MAF morphologies are problematic (e.g., dense 3D structures vs low-relief seafloor).

 Practitioners selecting tools need to know not just what is available, but what works for their specific habitat and resources. Consider strengthening Figures 3 & 7 by adding MAF-specific annotations or decision branches, for instance: "Suitable for these MAF types | Depth range | Water clarity requirements | Unsuitable morphologies." This would help practitioners quickly assess whether a recommended workflow is feasible for their specific context.

 2. a) The author states, " *critical for the spatial distribution of the MAF species are bathymetry and morphological parameters describing 3D microhabitats, the substrate, the direction and intensity of marine currents, the roughness of the sea bottom and its orientation, temperature, salinity and nutrients concentrations.*" - The Chimienti et al. (2019, 2020) citations provide good MAF-specific grounding for these variables. However, practitioners would benefit from guidance on variable prioritisation across different MAF types.

 Specifically:

 i) Are all nine variables equally critical for all MAF types? For example, do sponge grounds have the same drivers as coral reefs? Do polychaete reefs respond to different current regimes?

 ii) Which variables are non-negotiable vs. context-dependent? This helps practitioners with limited resources know where to focus monitoring effort (e.g., "Bathymetry + current intensity are essential; nutrients can be inferred from regional models").

 A brief table or section clarifying variable importance by MAF type (e.g., "Coral reefs: high priority on light, currents | Sponge grounds: high priority on depth, substrate") would strengthen practitioner guidance without requiring extensive new literature synthesis.

 b) Types of Data (species section) The claim that deep learning tools are 'becoming increasingly accessible' is supported by three recent tool papers (YOLO, RootPainter, MAIA). However, to substantiate this trend, consider citing a comprehensive review of AI adoption in marine ecology (e.g., Rubbens et al., 2023) that documents adoption timelines and actual uptake rates. Tool availability does not mean practitioner adoption; evidence of the trend would strengthen confidence.

 c) Biomass Distribution Modelling - Over-Reliance on Single Author Group - While De Clippele et al.'s (2021a, 2021b) approach is novel and valuable, the heavy reliance on this single work suggests the review may be incomplete. Are there other biomass mapping approaches from other research groups or taxonomic-specific applications?

 d) The section reviews available SDM algorithms generically. Practitioners need guidance on which algorithms are most effective for MAF-specific applications. Consider adding: which algorithms dominate MAF literature? Are there published comparative studies on MAF-specific performance? This would help practitioners select appropriate methods.

 3. The conclusion lists emerging technologies without substantive engagement. Readers learn that eDNA and ML exist, but not how they advance MAF mapping, what barriers remain, or why they matter. Consider either: (1) integrating these technologies into relevant methodology sections with concrete examples, or (2) removing speculative mentions

**Structural suggestion**:

 Consider adding a dedicated section on 'Emerging Technologies for MAF Mapping' (before the conclusion) to substantively discuss eDNA, ML, and sensor advances. This would:

 (1) Replace superficial mentions in the conclusion with detailed engagement (2) Honestly position these as 'pilot-stage' vs. 'production-ready' (3) Allow the conclusion to actually synthesise and recommend rather than introduce new topics

 This follows standard review structure: established -> emerging -> future. Currently, eDNA and ML appear only as afterthoughts in the final paragraph.

Is the review written in accessible language?

Yes

Are all factual statements correct and adequately supported by citations?

Partly

Are the conclusions drawn appropriate in the context of the current research literature?

Partly

Is the topic of the review discussed comprehensively in the context of the current literature?

Partly

Reviewer Expertise:

Marine habitat mapping

I confirm that I have read this submission and believe that I have an appropriate level of expertise to confirm that it is of an acceptable scientific standard, however I have significant reservations, as outlined above.

## References

[1] : Machine learning in marine ecology: an overview of techniques and applications. *ICES Journal of Marine Science* .2023;80(7) : 10.1093/icesjms/fsad100 1829-1853 10.1093/icesjms/fsad100

# Reviewer report: Reviewer response for version 2

**DOI:** 10.21956/openreseurope.23645.r69603

**Published:** 2026-02-27

**Authors:** Saskia Rühl

**Affiliations:** 1 Plymouth Marine Laboratory, Plymouth, UK

No further comments.

Is the review written in accessible language?

Yes

Are all factual statements correct and adequately supported by citations?

Yes

Are the conclusions drawn appropriate in the context of the current research literature?

Yes

Is the topic of the review discussed comprehensively in the context of the current literature?

Partly

Reviewer Expertise:

Benthic-pelagic exchanges, blue carbon mapping, benthic ecology, biological carbon cycle imaging.

I confirm that I have read this submission and believe that I have an appropriate level of expertise to confirm that it is of an acceptable scientific standard.

# Reviewer report: Reviewer response for version 1

**DOI:** 10.21956/openreseurope.22529.r59855

**Published:** 2025-09-26

**Authors:** Andrea Bryndum-Buchholz

**Affiliations:** 1 Memorial University, St John's, Canada

**SUMMARY**

 This review aims to provide a comprehensive overview and practical toolbox for mapping marine animal forests (MAFs) to support ecosystem understanding, conservation, and management. It synthesizes information on the types of data that can be mapped (biological and substrate), the platforms used to collect them (satellite, aerial, and underwater technologies such as sonar, acoustics, and cameras), and the analytical resources available (open databases, software, and modelling frameworks). Related modelling methods described include species, biomass, and community distribution modelling, as well as the growing use of machine learning and deep learning approaches, such as convolutional neural networks, to address data gaps and automate large-scale image analysis. By, among others, presenting a detailed overview table mapping tools, as well as a decision-support flow chart to help select appropriate tools based on project objectives, settings, and data availability, the study provides researchers, conservationists, and managers with a structured approach to mapping MAFs more effectively. After minor revisions, that largely relate to elaborating on certain details and streamlining the manuscript, this review will be a valuable contribution to the field. Please find below my major, more general comments, as well as more detailed, line-by-line comments.

**MAJOR COMMENTS (PLEASE ALSO REFER TO THE LINE-BY-LINE COMMENTS FOR SPECIFICS)**

- Language and audience: Overall, the manuscript is written in a clear way; however, the amount of detail provided between the sections differs quite a bit. Some are more general, others go into a lot of detail, using a lot of jargon.  For example, the sections “Sea-based mapping” and “Techniques: Mapping approaches” provide information that is rich in jargon and often misses explanations of information presented. Remember your audience. Some examples I suggest you focus on in your revision:
  - Page 10, last paragraph: First sentence is convoluted and difficult to follow. What does “their payload is modulable depending on each specific mission’s needs” mean? What would be a sufficiently longer period for AUVs, and why is this important to consider?
  - Page 11, first paragraph: What is the average time for divers under water in the mapping or sampling context? This information might be important to be able to compare the difference tools/techniques.
  - Page 11, “…making it ideal for applications in marine ecology, fisheries management, and environmental monitoring.”: Why and how? Please elaborate and explain.
  - Page 12, “…development of 3D models of underwater landscapes, enhancing visualization and analysis”: The “visualization and analysis” of what and how?
  - Page 12, second and third paragraph: Provide more context.
  - Page 14: Are there any drawbacks in using OBIS etc. users should know about? OBIS in the Northwest Atlantic, is known to have large data gaps, for example.
  - Page 16, first paragraph of “Species distribution modeling” section, “Each one has strengths and weaknesses, mostly around the learning curve and technical requirements”: This should not be left as is. What are those strengths and weaknesses? What is a learning curve and the technical requirements? If explaining these would go beyond the purpose of this paper, consider revising the overall manuscript to achieve a more general, les technical, wording.

Structure: There is some repetition between “Tools and technologies” and “Techniques: Mapping approaches”. How do you define tools and approaches in this manuscript? I suggest revise these sections with this in mind.

 Focus on common tools: There is a strong focus on commonly used tools in MAF mapping, which is valuable in its own right. What I was missing in this review was some insights into newer, innovative methods that can be used in the MAF mapping context, such as eDNA.

**LINE-BY-LINE COMMENTS**

 Abstract:

- Suggest using either “substrate” or “sedimentary” data.
- To improve flow of sentence, I suggest revising “…including space-based, air-based, and sea-based mapping tools.” by saying “…including space-, air-, and sea-based mapping tools.”
- Why detailing the sea-based mapping tools with “The latter include active acoustics, side-scan sonar, seismic reflection profiling, multibeam sonar, and underwater cameras.”, but not the other tools? Consider deleting.

Keywords:

- I suggest revising the list of keywords for search engine optimization. For example, use 2–4-word phrases, in other words avoid single words, as they can be too vague, and do not use full sentences or long phrases; Avoid using words from your title, since the title is already indexed by search engines, focus on keywords not present in it to maximize your reach. 

Introduction:

- Page 3: Keep the formatting of “Marine Animal Forest” consistent throughout the manuscript. Check for inconsistencies, like “marine animal forests”
- Is the Habitat Directive an EU directive? Maybe clarify.
- Page 3: “… currently, less than 25% of the ocean has been explored and remains unmapped (Mayer *et al.*, 2018), …” – Is there a more current reference for this statement?
- Page 3-4: Terms like “Littoral, Infralittoral, Shallow Circalittoral, Deep Circalittoral, Upper Bathyal, Lower Bathyal, and Abyssal.” are not capitalized.
- Page 4: I got a bit confused when the EUNIS habitat types “type A5.6” and “type A6.62” are mentioned in reference to Figure 1. However, on the figure, these types are not present. I suggest clarifying this either in the text or the figure.
- Page 4: “…used to develop full-coverage maps.” – Maps of what exactly? I suggest providing more detail here to improve clarity.
- Page 4: Provide a reference for the following statement: “Thanks to the development of remotely operated vehicles (ROVs), autonomous underwater vehicles (AUVs), submersibles, and drones, the spatial coverage and amount of image data of MAFs have skyrocketed in the last 25 years.”
- Page 4: While it is useful to provide information on the most common online data repositories,  PANGAEA, ( https://pangaea.de), Dryad ( https://datadryad.org), Zenodo ( https://zenodo.org/) and Figshare ( https://figshare.com), I was wondering if there are any concrete repositories from these resources for MAF data? If so, I suggest providing those instead, as those would be even more useful for the intended reader.
- Page 4: Paragraph starting with “While imagery collected by ROV, AUVs, and drones…” and ending with “…, especially in deep-sea and poorly studies marine environments”: I wonder if it would be effective for the introduction to add more details on how the “real biological samples” are being sampled. Also, what are “real” biological samples in comparison to “unreal” biological samples?
- Page 6: “Knowledge of the substrate is also essential for tracking changes due to climate change and human activities”. – How so? Please explain this connection a bit more, as it is not immediately clear why and how.
- Page 6: “…the size of an area that is to be mapped (meters, 10s, or 100s of kilometers),…” – Shouldn’t the units be m ^2^ and km ^2^? There is inconsistencies with these units throughout the manuscript.
- Page 7: “,…are used in almost 505 of the studies…” – please provide a timeframe here. Does this number refer to all studies ever or the ones reviewed in Nguyen et al. 2021?
- Page 7: “In 2021, twice as many papers ….” How is this sentence relevant for this section?
- Page 7: Provide a reference for sentence,  “Satellite imagery is especially useful for mapping large spatial areas…”.
- Page 7: “…drones and unmanned aerial systems (UAS), …” – these are technically the same.
- Page 13: Provide references for the first paragraph of the section “Multibeam sonar”.
- Page 13: Avoid introducing the acronyms again for ROVs and AUVs.
- Page 14: Provide references for the first paragraph of the section “Techniques: Mapping approaches”.
- Page 14: Can you provide links for the NASA Earthdata and or European Space
- Agency Climate Office?
- Page 14: Provide references for the last paragraph of this page (starting with “The use of unsupervised models…”)
- Page 15: What kind of “Clusters” are you referring to here?
- Page 16: Introduce acronyms for “IPCC” and “Marine Protected Areas”.
- Page 16: Use acronyms “MAFs” and “SDM” throughout this section once it has been introduced.

Figures:

- Figure 1: Suggest zooming in, as the other, unmapped areas, which are not part of the EUNIS project (?) are not relevant to show. This would also make the figure easier to read.
- Figure 2: Capitalize “boulders” in the read arrows to keep formatting consistent with the table below the triangles.
- Figure 3: Where does this figure come in? It is not referenced in the text. Please contextualise. Increase font size and overall image size. I also suggest to point to the flowchart figures more explicitly in the text, to highlight these as a decision tool.
- Figure 4: I am not sure what the purpose of this figure is in this manuscript. It does not give any information on water depth, as mentioned in the sentence where the figure is being referenced. It might also be a bit confusing to only show tropical coral reefs, as MAFs encompass so much more. Maybe delete or replace with a more representative figure?
- Figure 8: Increase font size and overall image size.

Is the review written in accessible language?

Yes

Are all factual statements correct and adequately supported by citations?

Partly

Are the conclusions drawn appropriate in the context of the current research literature?

Yes

Is the topic of the review discussed comprehensively in the context of the current literature?

Partly

Reviewer Expertise:

Marine ecology, marine conservation, MPAs, non-invasive monitoring of marine ecosystems.

I confirm that I have read this submission and believe that I have an appropriate level of expertise to confirm that it is of an acceptable scientific standard, however I have significant reservations, as outlined above.

# Response

**Published:** 2025-11-13

**Authors:** Laurence De Clippele

**Affiliations:** University of Glasgow College of Medical Veterinary and Life Sciences, Glasgow, Scotland, UK

-
  - Page 10, last paragraph: First sentence is convoluted and difficult to follow. What does “their payload is modulable depending on each specific mission’s needs” mean? What would be a sufficiently longer period for AUVs, and why is this important to consider?

**Response: **To address these comments, this section has now been changed to: Whereas there are some manned submersibles in use across the world, the number of unmanned vehicles largely outweighs the manned ones, mostly because of safety reasons and associated operational procedures and costs. The advantages of underwater platforms are that their design can be changed depending on each specific mission's needs and that they can operate at very short distances from the seafloor, thus enhancing data resolution. An inconvenience of these platforms is that they move at relatively low speeds, can struggle to hold position and can usually operate for only several hours a day, although longer continuous periods (e.g. several days-weeks) could be envisaged in the case of AUVs. Longer deployment times could allow for covering larger or more remote areas, or could allow for repeat surveys to understand changes in diel patterns.

-
  - Page 11, first paragraph: What is the average time for divers under water in the mapping or sampling context? This information might be important to be able to compare the difference tools/techniques.

**Response: **Information on time a diver can spent underwater has been added: However, the disadvantage if using divers is that they can only reach ~30 m water depth and that they can only spend a limited time underwater (~45-60 min, but 4-8 hours with a rebreather). Consequently, the area they can cover during surveys can be limited.

-
  - Page 11, “…making it ideal for applications in marine ecology, fisheries management, and environmental monitoring.”: Why and how? Please elaborate and explain.

**Response: **We agree that this sentence lacked the necessary detail to explain the utility of SSS across these three distinct domains. We have significantly revised and expanded this part of the paragraph to address the "why and how" for each field, using examples supported by the cited literature: This makes it particularly valuable for marine ecological research, as it enables the non-invasive detection of benthic habitats and biogenic structures that serve as refugia, feeding and nursery grounds for various species. In fisheries management, SSS-derived maps help delineate essential fish habitats, evaluate trawling impacts and monitor habitat recovery. In environmental monitoring, repeated SSS surveys can identify anthropogenic pressures such as dredging scars, marine litter accumulation or substrate alteration, thereby supporting long-term habitat condition assessments and conservation planning.

-
  - Page 12, “…development of 3D models of underwater landscapes, enhancing visualization and analysis”: The “visualization and analysis” of what and how?

**Response: **We agree that the role of 3D models required further clarification. We have revised the text to explain what is being visualized (geomorphological features, substrate heterogeneity, and habitat complexity) and how this is achieved—by improving our understanding of how seafloor morphology influences MAF distribution and habitat connectivity. The revised text now reads: (e) three-dimensional models, which, when integrated with multibeam bathymetry data, allow the development of 3D representations of underwater landscapes, enhancing the visualisation and analysis of geomorphological features, substrate heterogeneity, and habitat complexity. These models improve our understanding of how seafloor morphology influences species distribution, sediment transport, and habitat connectivity, providing a powerful tool for ecological and geological interpretation (e.g., Blondel, 2009; Canals et al., 2020; De Clippele et al., 2017; Dimitrov et al., 2019; Pillay et al., 2020).

-
  - Page 12, second and third paragraph: Provide more context.

**Response: **The paragraphs on the applications of Side Scan Sonar (SSS) have been expanded to provide additional ecological and methodological context relevant to MAF mapping. The revised text now explains how variations in backscatter intensity relate to substrate type and the attachment requirements of sessile MAF-forming species such as corals and sponges. It also clarifies the importance of automated image-classification approaches for processing large datasets typical of MAF habitats, such as deep-sea sponge grounds or patchy cold-water coral reefs. These revisions strengthen the ecological relevance and practical applicability of SSS mapping for understanding MAF distribution and habitat structure.

-
  - Page 14: Are there any drawbacks in using OBIS etc. users should know about? OBIS in the Northwest Atlantic, is known to have large data gaps, for example.

**Response: **Yes, OBIS has a coarse resolution, but is global. This sentence has now been added: Although OBIS has large data gaps, with a focus on data collected in western areas, it aims for global coverage.

-
  - Page 16, first paragraph of “Species distribution modeling” section, “Each one has strengths and weaknesses, mostly around the learning curve and technical requirements”: This should not be left as is. What are those strengths and weaknesses? What is a learning curve and the technical requirements? If explaining these would go beyond the purpose of this paper, consider revising the overall manuscript to achieve a more general, les technical, wording.

**Response: **Detailing this would be out of the scope of this paper, therefore this sentence has now been changed to the following to encourage the reader to read up on the different modelling approaches: Depending on e.g. the size of the dataset and the level of interpretability, or the accuracy of the model’s prediction needed, one may choose one approach over another. It is important for the user to understand the pros and cons of each modelling approach before applying or interpreting the results. Structure: There is some repetition between “Tools and technologies” and “Techniques: Mapping approaches”. How do you define tools and approaches in this manuscript? I suggest revise these sections with this in mind. With tools and technologies we refer to the platforms used to collect the data which is then used in analysis. The types of analysis are described in the “techniques: mapping approaches section. However, so it aligns better with the abstract and to avoid confusion, the latter section has now been renamed as “Analysis Approaches”. This has also been changed in the flowchart

 Focus on common tools: There is a strong focus on commonly used tools in MAF mapping, which is valuable in its own right. What I was missing in this review was some insights into newer, innovative methods that can be used in the MAF mapping context, such as eDNA. The conclusion section now also includes future directions. eDNA and ML are mentioned: “Future developments in sensor integration, machine learning (ML) algorithms, AI-driven classification, and advanced photogrammetric processing will further enhance its capabilities in underwater MAF mapping to classify coastal habitats with minimal or no field data collection (Liu 2022; Kvile et al. 2024b). However, both lower resolution and manual approaches remain having a key value, especially in unmapped or unexplored areas, which still comprise the majority of our oceans. Other novel approaches such as environmental DNA (eDNA) show promise for detecting the presence of species, and to study habitat-species interactions and may be used to map MAF in the future (Wilms et al. 2022; Bizzozzero et al., 2025).”

**LINE-BY-LINE COMMENTS**

 Abstract:

- Suggest using either “substrate” or “sedimentary” data.

**Response: **Sedimentary has been removed

- To improve flow of sentence, I suggest revising “…including space-based, air-based, and sea-based mapping tools.” by saying “…including space-, air-, and sea-based mapping tools.”

**Response: **Done, thank you.

- Why detailing the sea-based mapping tools with “The latter include active acoustics, side-scan sonar, seismic reflection profiling, multibeam sonar, and underwater cameras.”, but not the other tools? Consider deleting.

**Response: **Deleted

 Keywords:

- I suggest revising the list of keywords for search engine optimization. For example, use 2–4-word phrases, in other words avoid single words, as they can be too vague, and do not use full sentences or long phrases; Avoid using words from your title, since the title is already indexed by search engines, focus on keywords not present in it to maximize your reach. 

**Response: **Changed to: marine animal forest mapping, MAF mapping methods, review, species and substrate distribution modelling, marine mapping technologies, multibeam and side scan sonar, space and underwater water camera survey methods

 Introduction:

- Page 3: Keep the formatting of “Marine Animal Forest” consistent throughout the manuscript. Check for inconsistencies, like “marine animal forests”

**Response: **Now changed to marine animal forests or abbreviated as MAFs

- Is the Habitat Directive an EU directive? Maybe clarify.

**Response: **Yes, EU now added.

- Page 3: “… currently, less than 25% of the ocean has been explored and remains unmapped (Mayer *et al.*, 2018), …” – Is there a more current reference for this statement?

**Response: **Yes, announcement from seabed2030 states that 27.3% has now been mapped so this has been updated in the text

- Page 3-4: Terms like “Littoral, Infralittoral, Shallow Circalittoral, Deep Circalittoral, Upper Bathyal, Lower Bathyal, and Abyssal.” are not capitalized.

**Response: **This has now been corrected.

- Page 4: I got a bit confused when the EUNIS habitat types “type A5.6” and “type A6.62” are mentioned in reference to Figure 1. However, on the figure, these types are not present. I suggest clarifying this either in the text or the figure.

**Response: **The figure doesn’t show all of the habitat types. As indicated by the legend’s vertical navigation bar. To avoid confusion, the caption has now been changed to Figure 1. Map of predicted seabed habitat types (EUNIS 2019 classification) across European waters as visualised in the EMODnet Map Viewer (Vasquez et al., 2021). Colour-coded categories correspond to habitat types listed in the legend. Source: European Marine Observation and Data Network (EMODnet). Note that, as indicated by the horizontal navigation bar in the legend, not all habitat types are listed in the legend.

- Page 4: “…used to develop full-coverage maps.” – Maps of what exactly? I suggest providing more detail here to improve clarity.

**Response: **To clarify this sentence has been changed to: “As biological data are often limited, species and habitat distribution modelling approaches are increasingly being used to develop full-coverage MAF maps, i.e. without spatial data gaps”

- Page 4: Provide a reference for the following statement: “Thanks to the development of remotely operated vehicles (ROVs), autonomous underwater vehicles (AUVs), submersibles, and drones, the spatial coverage and amount of image data of MAFs have skyrocketed in the last 25 years.”

**Response: **There is no specific review that shows this, however we have added a reference that discusses the history and evolution of the use of ROVs and AUVs and some of the challenges associated with increase image data. We edited the final part of the sentence to “has drastically increased”

- Page 4: While it is useful to provide information on the most common online data repositories,  PANGAEA, ( https://pangaea.de), Dryad ( https://datadryad.org), Zenodo ( https://zenodo.org/) and Figshare ( https://figshare.com), I was wondering if there are any concrete repositories from these resources for MAF data? If so, I suggest providing those instead, as those would be even more useful for the intended reader.

**Response: **These repositories are indeed used for a wide variety of data types associated with different papers from different fields. The repositories are mentioned here as not only can it help the reader to find data, but also deposit data. To make clearer that the reader would need to look for papers, we have now added the following sentence:  When searching for relevant data, researchers should review both the repository entries and the corresponding publications, as data are frequently linked or described within related papers.

- Page 4: Paragraph starting with “While imagery collected by ROV, AUVs, and drones…” and ending with “…, especially in deep-sea and poorly studies marine environments”: I wonder if it would be effective for the introduction to add more details on how the “real biological samples” are being sampled. Also, what are “real” biological samples in comparison to “unreal” biological samples?

**Response: **As per the other reviewer’s comment the word “real” has been changed with “physical”. The paragraph now includes this statement “Physical samples can e.g. be collected with ROVs, trawls, grab-and boxcore samplers, and enable detailed taxonomic analysis, including microscopic and genetic examination, which is crucial for confirming species identity, identifying cryptic species, and refining image-based identification protocols”

- Page 6: “Knowledge of the substrate is also essential for tracking changes due to climate change and human activities”. – How so? Please explain this connection a bit more, as it is not immediately clear why and how.

**Response: **To clarify,  this sentence has been changed to: Knowledge of the substrate is also essential to understand how the substrate composition and e.g. coastal flood risk, may change as a consequence of climate change and human activities (Trifonova et al. 2015).

- Page 6: “…the size of an area that is to be mapped (meters, 10s, or 100s of kilometers),…” – Shouldn’t the units be m ^2^ and km ^2^? There is inconsistencies with these units throughout the manuscript.

**Response: **Correct, thank you. This has now been changed and checked throughout.

- Page 7: “,…are used in almost 505 of the studies…” – please provide a timeframe here. Does this number refer to all studies ever or the ones reviewed in Nguyen et al. 2021?

**Response: **Only the ones reviewed by Nguyen. This has now been changed to: “In the review conducted by Nguyen et al. (2021) satellites, as a tool to map tropical coral reefs, are used in almost 50% of the studies and are recommended by the Coral Reef Expert Group for habitat MAF mapping and change detection on a broad scale (González-Rivero et al. 2020).

- Page 7: “In 2021, twice as many papers ….” How is this sentence relevant for this section?

**Response: **It  indicates that the popularity of this tool has increased over time. Changed to: The popularity of using satellite data has increased, especially in recent years. E.g. in 2021, twice as many papers were published in the two years prior compared to in the last ten years (Nguyen et al. 2021).

- Page 7: Provide a reference for sentence,  “Satellite imagery is especially useful for mapping large spatial areas…”.

**Response: **(Palandro et al. 2003) reference added.

- Page 7: “…drones and unmanned aerial systems (UAS), …” – these are technically the same.

**Response: **Removed “drones and”

- Page 13: Provide references for the first paragraph of the section “Multibeam sonar”.

**Response: **Menandos and Bastos (2020) has been added

- Page 13: Avoid introducing the acronyms again for ROVs and AUVs.

**Response: **Thanks, this has now been corrected.

- Page 14: Provide references for the first paragraph of the section “Techniques: Mapping approaches”.

**Response: **The following reference have now been added: (e.g. Morato et al. 2020, Schumacher et al., 2022, Vinha et al. 2025)

- Page 14: Can you provide links for the NASA Earthdata and or European Space

**Response: **Agency Climate Office? Now added: NASA Earthdata (https://www.earthdata.nasa.gov/) and/or European Space Agency Climate Office (https://climate.esa.int) (providing satellite imagery of environmental conditions.

- Page 14: Provide references for the last paragraph of this page (starting with “The use of unsupervised models…”)

**Response: **Calvert et al. 2015 has been added

- Page 15: What kind of “Clusters” are you referring to here?

**Response: **Paragraph has been changed to: In unsupervised habitat characterisation, clusters represent statistically similar groups of environmental or acoustic features that may correspond to distinct habitat types. These clusters are subsequently interpreted and validated using ground-truth data, most commonly underwater imagery, which provides wider spatial coverage than physical samples collected by drags or grabs. Additionally, it is a non-invasive method, particularly suitable for complex areas such as rocky bottoms or slopes, without causing significant damage to the ecosystem.  

- Page 16: Introduce acronyms for “IPCC” and “Marine Protected Areas”.

**Response: **Intergovernmental Panel on Climate Change added and (MPA) acronym now

- Page 16: Use acronyms “MAFs” and “SDM” throughout this section once it has been introduced.

**Response: **SDM is introduced at the start of section 4.3. MAF is introduced in section 2.1 Figures:

- Figure 1: Suggest zooming in, as the other, unmapped areas, which are not part of the EUNIS project (?) are not relevant to show. This would also make the figure easier to read.

**Response: **Unfortunately it was not possible to zoom in more without consequently losing parts of the area that has been characterised.

- Figure 2: Capitalize “boulders” in the read arrows to keep formatting consistent with the table below the triangles.

**Response: **This has now been edited as suggested.

- Figure 3: Where does this figure come in? It is not referenced in the text. Please contextualise. Increase font size and overall image size. I also suggest to point to the flowchart figures more explicitly in the text, to highlight these as a decision tool.

**Response: **This figure is referenced, in section 3. Tools and technologies. This sentsence has now been added as well: A flowchart is provided in Figure 3 to help a user decide on what tool to use (Figure 3). The font size is now also increased

- Figure 4: I am not sure what the purpose of this figure is in this manuscript. It does not give any information on water depth, as mentioned in the sentence where the figure is being referenced. It might also be a bit confusing to only show tropical coral reefs, as MAFs encompass so much more. Maybe delete or replace with a more representative figure?

**Response: **Sentence has now been edited to make clearer why this figure was included: For instance, satellite imagery represents a powerful tool to map the extent of shallow tropical coral reefs, data which has been used to create the Allen Coral Atlas (Figure 4). (Lyons et al. 2024).

- Figure 8: Increase font size and overall image size.

**Response: **Font and image size have now been increased

# Reviewer report: Reviewer response for version 1

**DOI:** 10.21956/openreseurope.22529.r60645

**Published:** 2025-09-26

**Authors:** Saskia Rühl

**Affiliations:** 1 Plymouth Marine Laboratory, Plymouth, UK

**General comments:**

 The article reviews methods and tools for surveying benthic habitats, and mapping Marine Animal Forests (MAFs), making recommendations and providing practical tools to aid researchers in choosing the correct methods.

 It is overall a comprehensive review worthy of publication, though the focus on MAFs could be stronger throughout, as many tools and methods are, by themselves, only suitable for more general habitat mapping purposes. The interchangeable way in which the review treats MAF mapping and habitat mapping is misleading, but can be amended through some rephrasing throughout (see examples in detailed comments below). Additionally, more examples of different kinds of MAFs would be useful, to avoid focussing almost exclusively on coral reefs as examples, which could be misleading to readers who are not that familiar with MAFs. Especially the types of MAF for which the tools described in this report are unsuitable, should be included to give the full picture. The headings & sub-heading could be divided into more detailed subsections (example in detailed comments).

 In my opinion, some re-writing and adding to the text will be necessary, before indexing,

**Detailed comments:**

 (Note that due to the lack of line numbers, feedback is structured based on the page numbers of the pages of the file, once exported as a pdf, with pages one and two containing the title page, abstract and author details)

1. Page 4, Right column, Paragraph 1: This paragraph should be re-framed as the database data will have been collected using some of the same methods, not instead of using them. Something along the lines of “Instead of collecting one’s own data, it is possible to source pre-collected data from databases such as…”; Also, add a caution here as well on how reliable those data are, how well documented, and how much is measured vs. inferred / modelled.
2. Page 4, Right column, Paragraph 2: Increased accessibility is not a challenge, but a solution.
3. Page 4, Right column, Paragraph 3: “…distributions real biological sampling”, replace “real” with “physical” or “traditional”.
4. Page 4, Right column, Paragraph 3: Acoustic sampling of biological variables is mentioned, but no detail is given. Please elaborate on this, if relevant, and if not, be more specific when introducing the method earlier on.
5. Page 5, Right column, Paragraph 1: Insert a note on carbon tracking and climate smart spatial planning, identifying deposition areas. Also add details on how grain size is measured, in-situ or through lab-based particle size analysis. If the methods for biota determination are detailed, then the methods of identifying substrate types should be similarly detailed.
6. Page 5, Right column, Paragraph 2: See previous comment, more methods details would be good.
7. Page 6, Left column, Paragraph 2: See comment 1, regarding cautions on data origins and reliability.
8. Page 8, Right column, Paragraph 1: The section preceeding this gives the impression, that remote sensing techniques are only good for coral mapping. If the paper’s purpose is to make recommendations for MAF in general, more examples of different types of MAF should be given.
9. Page 9, Right column, Paragraph 2: “…air based mapping presents several challenges.” seems counterintuitive. Do the authors mean that air-based mapping faces these challenges?
10. Page 9, Right column, Paragraph 2: This paragraph also contains factors that could be challenging for space-based mapping as well, and weren’t mentioned in that section. Related to this, in the sentence “As for satellite-based images, air-based methods can only be used…”, the wording should be along the lines of “Similarly to satellite-based images, air-based methods can only be used…”.
11. Page 9, Right column, Paragraph 3: This is a nice conclusion paragraph, but I would recommend to be consistent and do one for each of the “-based mapping” sections.
12. Page 9, Right column, Paragraph 3: The mention of “artificial intelligence (AI)/machine learning (ML)” is redundant, as ML is a kind of AI.
13. Page 10, Left column, Paragraph 2: the end of this paragraph is a good example of when habitat mapping is mentioned in a situation describing substrate mapping techniques, which could be misleading the reader into thinking that this by itself is a valid MAF mapping approach, as mentioned in the general comments.
14. Page 11, Left column, Paragraph 1: Clarification is needed when describing that “…they can check in situ if a given object is hard or soft, …” Does this refer to substrate-types, or do the authors mean to say that divers can distinguish living from non-living objects?
15. Page 11, Left column, Paragraph 1: There should be a note at the end of this paragraph on the cons of using divers for mapping, to be consistent with how the other methods were described.
16. Page 11, Left column, Paragraph 2: This paragraph appears disjointed from the others, and needs to be tied-in better. I suggest the authors include the reasoning behind their choice of tools and methods for MAF mapping purposed in each of the previous sections, to lead into how this conclusion was reached.
17. Page 11, Left column, between paragraphs 2 and 3: I’d suggest adding a sub-section heading here, “Mapping tools” or similar. The authors should also consider whether the placement of this whole section on tools might not be more fitting before the section on platforms, as the tools are mentioned throughout the platform section already. Swapping the two might reduce repetition.
18. Page 11, Left column, Paragraph 3: The Side Scan Sonar profiling section is one of the examples of MAFs never even being mentioned. The paper would be more cohesive if the mention of MAFS ran through it as a red thread, rather than only framing it at the start and end. See comment on the confusing terminology use of habitat mapping vs MAF mapping, in general comments.
19. Page 11, Right column, Paragraph 1: Add mention of weaknesses of the method.
20. Page 12, Right column, Paragraph 2: Example of an exclusive focus on corals as an example of MAFs. Using more varied examples would make a stronger case for the utility of the methods and tools described here.
21. Page 14, Left column, Paragraph 4: Include a note on camera use for MAF mapping purposes specifically. How widely is this used?
22. Page 14, Right column, Paragraph 2: Some of the biogeochemical variables mentioned here are highly spatially variable, so the data sources or sampling/sensing methods should be mentioned. Where is the data coming from? This is also the first mention of using biogeochemical data variables for MAF mapping, and should probably have been at least mentioned earlier in the introduction.
23. Page 16, Left column, Paragraph 4: This is the first explicit mention of Marine Protected Areas, and while the acronym MPA was used in the caption of Figure 6 already, the acronym is not introduced together with the written-out version as it should be.
24. Page 16, Right column, Paragraph 4: The section on community distribution modelling is another that doesn’t mention MAFs explicitly.

Is the review written in accessible language?

Yes

Are all factual statements correct and adequately supported by citations?

Yes

Are the conclusions drawn appropriate in the context of the current research literature?

Yes

Is the topic of the review discussed comprehensively in the context of the current literature?

Partly

Reviewer Expertise:

Benthic-pelagic exchanges, blue carbon mapping, benthic ecology, biological carbon cycle imaging.

I confirm that I have read this submission and believe that I have an appropriate level of expertise to confirm that it is of an acceptable scientific standard, however I have significant reservations, as outlined above.

# Response

**Published:** 2025-11-13

**Authors:** Laurence De Clippele

**Affiliations:** University of Glasgow College of Medical Veterinary and Life Sciences, Glasgow, Scotland, UK

The manuscript has been edited and/or rewritten as suggested by the reviewer’s comments. We have now not interchangeably referred to MAF and habitat mapping to avoid confusion. Where needed and possible more examples from literature have been provided. Headings and subheadings have also been changed as suggested.  

**Detailed comments:**

  

1. Page 4, Right column, Paragraph 1: This paragraph should be re-framed as the database data will have been collected using some of the same methods, not instead of using them. Something along the lines of “Instead of collecting one’s own data, it is possible to source pre-collected data from databases such as…”; Also, add a caution here as well on how reliable those data are, how well documented, and how much is measured vs. inferred / modelled.

**Response: **This section is now adjusted to : “One of the challenges of using approaches based on video and image analysis is that high-resolution taxonomic identification is not always possible. However, there are different standardised annotation protocols for image-based identifications that can be followed. Some of these protocols include the CATAMI classification scheme (Althaus et al. 2015), SmarTaR-ID (Howell et al. 2019) or the use of Open nomenclature (ON) signs (Horton et al. 2021).  Instead of collecting one’s own data, it is possible to source pre-collected data from databases such as: Ocean Biogeographic Information System (OBIS) (https://obis.org), the Global Biodiversity Information Facility (GBIF) (https://www.gbif.org), NOAA’s Deep-sea Coral Portal (https://deepseacoraldata.noaa.gov), UNEP Global Distribution of Cold-water corals (Freiwald et al., 2021; https://data-gis.unep-wcmc.org) and Online data repositories such as PANGAEA (https://pangaea.de), Dryad (https://datadryad.org), Zenodo (https://zenodo.org/) and Figshare (https://figshare.com).  Note that while some databases included raw data, some may contain modelled or inferred data and would therefore need to be interpreted with care.”

1. Page 4, Right column, Paragraph 2: Increased accessibility is not a challenge, but a solution.

**Response: **Paragraph changed to: “Another challenge is related to the analysis of large image datasets. Deep learning approaches offers a solution, such as the use of convolutional neural networks, are becoming increasingly accessible for benthic ecologists. Examples of user-friendly tools are YOLO (Piechaud and Howell 2022), RootPainter (Clark et al. 2024), and MAIA in BIIGLE (Cuvelier et al. 2024).”

1. Page 4, Right column, Paragraph 3: “…distributions real biological sampling”, replace “real” with “physical” or “traditional”.

**Response: **This has been replaced with the word “physical” as suggested by the reviewer. This has also been edited in the final sentence of this paragraph.

1. Page 4, Right column, Paragraph 3: Acoustic sampling of biological variables is mentioned, but no detail is given. Please elaborate on this, if relevant, and if not, be more specific when introducing the method earlier on.

**Response: **Acoustic sampling is not described in the paragraph, so the authors are unclear what this comment by the reviewer means.

1. Page 5, Right column, Paragraph 1: Insert a note on carbon tracking and climate smart spatial planning, identifying deposition areas. Also add details on how grain size is measured, in-situ or through lab-based particle size analysis. If the methods for biota determination are detailed, then the methods of identifying substrate types should be similarly detailed.

**Response: **In response to the reviewer’s comment, we have added a new paragraph at the end of the Grain size section (2.2.2, Substrate) to address the role of fine-grained sediments in carbon tracking and climate-smart spatial planning. The revision explains how silt and clay fractions act as natural carbon sinks, supporting the identification of depositional areas and contributing to long-term carbon sequestration. Relevant references were incorporated to substantiate these concepts (Bao et al., 2019; Dahl et al., 2016; Guo et al., 2021; Legge et al., 2020; Müller et al., 2025; Wang et al., 2024; Zhang et al., 2024). Additionally, a separate paragraph was introduced to highlight the methodological importance of precise sediment characterisation for engineering and habitat-mapping tasks. This section clarifies that sediment composition is determined in specialised laboratories using standardised techniques (sieving, hydrometer, pipette, laser granulometry) and processed with the GRADISTAT software (Blott & Pye, 2001), ensuring analytical consistency and reliability for marine habitat mapping.

1. Page 5, Right column, Paragraph 2: See previous comment, more methods details would be good.

**Response: **This has now been added: “The relationship between grain size and sedimentary processes also extends beyond physical classification, linking substrate characteristics to broader ecological and biogeochemical functions of the seabed. Beyond its geomorphological relevance, grain size plays a key role in carbon tracking and climate-smart spatial planning. Fine-grained sediments, such as silt and clay, are recognised as effective sinks for organic carbon due to their high surface area, low permeability and capacity to protect organic matter from remineralisation (Bao et al., 2019; Guo et al., 2021; Müller et al., 2025). In contrast, coarse sediments are typically associated with high hydrodynamic energy and limited carbon retention capacity (Dahl et al., 2016). Mapping depositional environments dominated by fine-grained sediments is therefore essential for identifying natural carbon sinks and understanding the spatial variability of organic carbon burial (Legge et al., 2020; Wang et al., 2024). Integrating grain-size and carbon-content data within marine habitat mapping frameworks enables a more comprehensive assessment of seabed functions, supporting climate-smart spatial planning and the prioritisation of areas contributing to long-term carbon sequestration and ecosystem resilience (Müller et al., 2025; Zhang et al., 2024). Grain-size analysis represents a fundamental step in characterising marine substrates, as engineering projects and habitat-mapping tasks require high analytical precision and reliability in determining sediment composition and texture. Such analyses are conducted in specialised sediment laboratories, where standardised methodologies and controlled conditions ensure consistency of results. Laboratory techniques commonly include wet and dry sieving for coarser fractions, hydrometer and pipette analyses for fine-grained particles, and laser granulometry for detailed particle-size spectra. The processing, statistical treatment, and graphical visualisation of these datasets are frequently performed using the Excel-based software GRADISTAT, developed by Blott and Pye (2001), which enables standardised and reproducible sediment classification across marine and terrestrial environments. These laboratory-based approaches provide the quantitative foundation for accurate interpretation of depositional dynamics, substrate heterogeneity, and sediment-associated biogeochemical processes.”  

1. Page 6, Left column, Paragraph 2: See comment 1, regarding cautions on data origins and reliability.

**Response: **Paragraph edited to: International and national databases compile seabed sediment data to provide standardised information. A key example is the EMODnet Geology Seabed Substrate Database (https://emodnet.ec.europa.eu/en/geology), which provides seabed substrate data for European seas based on modelled data.

1. Page 8, Right column, Paragraph 1: The section preceeding this gives the impression, that remote sensing techniques are only good for coral mapping. If the paper’s purpose is to make recommendations for MAF in general, more examples of different types of MAF should be given.

**Response: **Changed to: While this approach has been shown useful for shallow shellfish beds  (Nieuwhof et al. 2015) , tropical coral reefs can extend beyond 20 m depth, and therefore, maps produced with satellite imagery may underestimate the true extent of habitats such as coral reefs that grow at deeper depths (Liaño-Carrera et al. 2019; Eyal et al. 2021; Galbraith et al. 2022).

1. Page 9, Right column, Paragraph 2: “…air based mapping presents several challenges.” seems counterintuitive. Do the authors mean that air-based mapping faces these challenges?

**Response: **Indeed, now changed to: Despite its many benefits, air-based mapping also faces several challenges

1. Page 9, Right column, Paragraph 2: This paragraph also contains factors that could be challenging for space-based mapping as well, and weren’t mentioned in that section. Related to this, in the sentence “As for satellite-based images, air-based methods can only be used…”, the wording should be along the lines of “Similarly to satellite-based images, air-based methods can only be used…”.

**Response: **The same challenges were mentioned but this was indeed not as clear. We have therefore changed the sentence in the satellite section to: Areas that have a lot of cloud cover may have clouds “blocking” the view, and light attenuation in water due to absorption (e.g. when there is higher turbidity or at deeper depth) and scattering (e.g. due to wave action) results in satellites often being unable to map accurately and/or below 20 m depth. While this approach has been shown useful for shallow shellfish beds  (Nieuwhof et al. 2015) Similarly to satellite-based images, airborne methods can only be used to map relatively shallow systems (~20 m depth).

1. Page 9, Right column, Paragraph 3: This is a nice conclusion paragraph, but I would recommend to be consistent and do one for each of the “-based mapping” sections.

**Response: **Conclusions are integrated in each of the sections, but to avoid inconsistency in writing style this paragraph has now been moved to the conclusion + future direction section.

1. Page 9, Right column, Paragraph 3: The mention of “artificial intelligence (AI)/machine learning (ML)” is redundant, as ML is a kind of AI.

**Response: **We have removed artificial intelligence (AI) from this sentence

1. Page 10, Left column, Paragraph 2: the end of this paragraph is a good example of when habitat mapping is mentioned in a situation describing substrate mapping techniques, which could be misleading the reader into thinking that this by itself is a valid MAF mapping approach, as mentioned in the general comments.

**Response: **Changed to: Active acoustic (e.g. seismic and multibeam sonar) and image-based tools are attached to sea-based mapping platforms to collect depth or backscatter information of habitats at local and regional scales.

1. Page 11, Left column, Paragraph 1: Clarification is needed when describing that “…they can check in situ if a given object is hard or soft, …” Does this refer to substrate-types, or do the authors mean to say that divers can distinguish living from non-living objects?

**Response: **This refers to non-living objects. This is now changed to: “For example, they can check in situ if a given substrate is hard or soft, a property that is often not obvious from imagery alone”

1. Page 11, Left column, Paragraph 1: There should be a note at the end of this paragraph on the cons of using divers for mapping, to be consistent with how the other methods were described.

**Response: **The cons are described here already, i.e. they can only go to certain depths and can only stay in the water for a limited amount of time. However, to clarify this, this is now changed to: “However, the disadvantage if using divers is that they can only reach ~30 m water depth and that they can only spend a limited time underwater. Consequently, the area they can cover during surveys is limited.

1. Page 11, Left column, Paragraph 2: This paragraph appears disjointed from the others, and needs to be tied-in better. I suggest the authors include the reasoning behind their choice of tools and methods for MAF mapping purposed in each of the previous sections, to lead into how this conclusion was reached.

**Response: **We agree with the reviewer's comment that this paragraph is a bit disjointed and decided to move it as it aligned better as a continuation of the first paragraph of this section.

1. Page 11, Left column, between paragraphs 2 and 3: I’d suggest adding a sub-section heading here, “Mapping tools” or similar. The authors should also consider whether the placement of this whole section on tools might not be more fitting before the section on platforms, as the tools are mentioned throughout the platform section already. Swapping the two might reduce repetition.

**Response: **Per the reviewers' suggestion, the two sections have now been swapped, and two subheadings have been added: “Sea-based mapping platforms” and “sea-based mapping tools”

1. Page 11, Left column, Paragraph 3: The Side Scan Sonar profiling section is one of the examples of MAFs never even being mentioned. The paper would be more cohesive if the mention of MAFS ran through it as a red thread, rather than only framing it at the start and end. See comment on the confusing terminology use of habitat mapping vs MAF mapping, in general comments.

**Response: **We have now replaced “habitat mapping” with “MAF mapping” throughout for consistency

1. Page 11, Right column, Paragraph 1: Add mention of weaknesses of the method.

**Response: **The paragraph on Side Scan Sonar profiling was expanded to include the main methodological limitations of the SSS technique, such as lack of bathymetric and subsurface information, positional inaccuracies, acoustic shadows, resolution trade-offs, and operator dependency. These aspects were supported by recent high-impact references (Grządziel 2023; Smith et al. 2023; von Rönn et al. 2019; Zhao et al. 2018; Shang et al. 2019; Helferty & Johnson 2009).

1. Page 12, Right column, Paragraph 2: Example of an exclusive focus on corals as an example of MAFs. Using more varied examples would make a stronger case for the utility of the methods and tools described here.
2. Page 14, Left column, Paragraph 4: Include a note on camera use for MAF mapping purposes specifically. How widely is this used?

**Response: **CWC mounds are the best example of MAF imaging by seismic reflection profiling at variable subseafloor depths. CWC mounds include a large variety of other organisms, not just corals. Other types of MAFs are generally extremely hard to identify in seismic reflection profiles, with, perhaps, the exception of bank oysters. Bryozoans and other molluscs can also form bioconstructions potentially identifiable in seismic reflection profiles, but the authors are not aware of undisputable seismic images of them. Therefore this section has been reworded as: “Seismic reflection profiling. Seismic reflection profiling is a widely used tool to image the subseafloor at shallow (1-10’s of meters scale), intermediate (10’s to a few 100’s of meters scale) and large depths (several 100’s of meters to kilometres scale) (Crutchley & Kopp, 2018). This method can be used to reveal information on the formation and history of certain marine animal forests, as best illustrated by cold-water coral carbonate mounds, which encompass a large variety of organisms other than corals.”

1. Page 14, Right column, Paragraph 2: Some of the biogeochemical variables mentioned here are highly spatially variable, so the data sources or sampling/sensing methods should be mentioned. Where is the data coming from? This is also the first mention of using biogeochemical data variables for MAF mapping, and should probably have been at least mentioned earlier in the introduction.

**Response: **More detail on where the data comes from has now been added and this paragraph was changed to: “Among the most common environmental parameters, critical for the spatial distribution of the MAF species are bathymetry and morphological parameters describing 3D microhabitats, the substrate, the direction and intensity of marine currents, the roughness of the sea bottom and its orientation, temperature, salinity and nutrients concentrations (e.g. Chimienti et al. 2019, 2020). Terrain variables, such as roughness, orientation, depth, bathymetric positioning index and slope, can be calculated from bathymetry data in R or GIS (e.g. De Clippele et al. 2017; Ramiro-Sánchez et al., 2019). The direction and intensity of marine currents can be measured with current speed sensors and acoustic doppler current profilers (ADCPs). Temperature, salinity and depth can be measured with Conductivity, Temperature and Depth (CTD) sensors. Finally, nutrient concentrations can be measured with sediment traps, Chl-a sensors or even satellite data (e.g. Vad et al., 2025). Information on the substrate can be either extracted from images/videos, from backscatter data or from physical samples. Much relevant data is now also available as part of open-access databases, such as GEBCO bathymetry (GEBCO, 2024) (providing a global bathymetric grid). Copernicus Marine Service (providing different physical and chemical oceanographical data products), Bio-Oracle (Tyberghein et al. 2012; Assis et al. 2018, 2024) (providing current and future physical and chemical oceanographic datasets), NASA Earthdata and or European Space Agency Climate Office (providing satellite imagery of environmental conditions” This sentence has also been added to the introduction section 2.2: “This data is then often analysed alongside environmental data (e.g., terrain variables, temperature, salinity, current speed and orientation) to understand what drives spatial patterns.”

1. Page 16, Left column, Paragraph 4: This is the first explicit mention of Marine Protected Areas, and while the acronym MPA was used in the caption of Figure 6 already, the acronym is not introduced together with the written-out version as it should be.

**Response: **The acronym has now been added when marine protected areas are mentioned for the first time.

1. Page 16, Right column, Paragraph 4: The section on community distribution modelling is another that doesn’t mention MAFs explicitly.

**Response: **To address this comment, the first sentence has now been changed to : “Instead of modelling the distribution of species individually, it is also possible to model the distribution of species assemblages or communities associated with MAFs (Vinha et al. 2025).”
